# MicroRNA-31 negatively regulates peripherally derived regulatory T-cell generation by repressing retinoic acid-inducible protein 3

**DOI:** 10.1038/ncomms8639

**Published:** 2015-07-13

**Authors:** Lingyun Zhang, Fang Ke, Zhaoyuan Liu, Jing Bai, Jinlin Liu, Sha Yan, Zhenyao Xu, Fangzhou Lou, Hong Wang, Huiyuan Zhu, Yang Sun, Wei Cai, Yuanyuan Gao, Qun Li, Xue-Zhong Yu, Youcun Qian, Zichun Hua, Jiong Deng, Qi-Jing Li, Honglin Wang

**Affiliations:** 1 Shanghai Institute of Immunology, Key Laboratory of Cell Differentiation and Apoptosis of Chinese Ministry of Education, Shanghai Jiao Tong University School of Medicine (SJTU-SM), Shanghai 200025, China; 2 Shanghai Institute of Hypertension, Ruijin Hospital, Shanghai Jiao Tong University School of Medicine (SJTU-SM), Shanghai 200025, China; 3 Department of Microbiology and Immunology, Medical University of South Carolina, Charleston, South Carolina 29425, USA; 4 Institute of Health Sciences, Shanghai Institutes for Biological Sciences, Chinese Academy of Sciences/Shanghai Jiao Tong University School of Medicine (SJTU-SM), Shanghai 200025, China; 5 State Key Laboratory of Analytical Chemistry for Life Science, Nanjing University, Nanjing 210093, China; 6 Key Laboratory of Cell Differentiation and Apoptosis of Minister of Education, Shanghai Jiao Tong University School of Medicine (SJTU-SM), Shanghai 200025, China; 7 Department of Immunology, Duke University Medical Center, Durham, North Carolina 27710, USA; 8 Shanghai Key Laboratory for Tumor Microenvironment and Inflammation, Shanghai Jiao Tong University School of Medicine (SJTU-SM), Shanghai 200025, China

## Abstract

Peripherally derived regulatory T (pT_reg_) cell generation requires T-cell receptor (TCR) signalling and the cytokines TGF-β1 and IL-2. Here we show that TCR signalling induces the microRNA miR-31, which negatively regulates pT_reg_-cell generation. miR-31 conditional deletion results in enhanced induction of pT_reg_ cells, and decreased severity of experimental autoimmune encephalomyelitis (EAE). Unexpectedly, we identify Gprc5a as a direct target of miR-31. Gprc5a is known as retinoic acid-inducible protein 3, and its deficiency leads to impaired pT_reg_-cell induction and increased EAE severity. By generating *miR-31* and *Gprc5a* double knockout mice, we show that miR-31 promotes the development of EAE through inhibiting Gprc5a. Thus, our data identify miR-31 and its target Gprc5a as critical regulators for pT_reg_-cell generation, suggesting a previously unrecognized epigenetic mechanism for dysfunctional T_reg_ cells in autoimmune diseases.

## Abstract (web summary)

Peripherally derived regulatory T cells (pT_reg_) exhibit immunosuppressive capacity. Here, the authors show that microRNA-31 acting through inhibiting its direct target Gprc5a negatively regulates pT_reg_ generation and promotes the development of experimental autoimmune encephalomyelitis.

## Graphical abstract

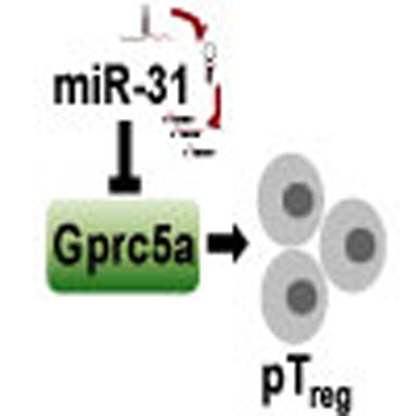

T cells serve as a central cellular player in adaptive immunity, and their activation and differentiation are elicited by signals from T-cell receptor (TCR), co-stimulatory receptors and various cytokines^1^. Once activated by an antigen, naive CD4^+^ T cells proliferate and differentiate into various T helper (T_H_) cell subsets, including T_H_1, T_H_2, T_H_17 and regulatory T (T_reg_) cells, that release different cytokines and exhibit distinct effector functions^2^. Besides their critical role in driving immune responses against infections, T_H_1 and T_H_17 cells participate in the pathogenesis of autoimmune inflammatory diseases, such as experimental autoimmune encephalomyelitis (EAE)^3^. Moreover, naive T cells differentiate into T_reg_ cells exhibiting immunosuppressive capacity, and the transcriptional factor FoxP3 controls their development and fucntion^4^,^5^. According to their origins, T_reg_ cells are divided into thymus-derived T_reg_ (tT_reg_) cells derived from the thymus, peripherally derived regulatory T (pT_reg_) cells generated out of the thymus under various inductive signals, and *in vitro*-induced regulatory T (iT_reg_) cells^6^,^7^. It is now clear that naive CD4^+^ T cells sorted as FoxP3^−^ in the thymus possess full potential to differentiate into pT_reg_ cells, thus are potential targets for therapeutic interventions for chronic inflammatory diseases^7^,^8^. Independent on thymus, pT_reg_ cells differentiate in secondary lymphoid organs and tissues, and require TCR signalling and the cytokines TGF-β and IL-2 (ref. 7), and only a low antigen dose of a high-affinity TCR ligand is optimal to generate a persistent population of pT_reg_ cells *in vivo*^9^.

So far, dysfunctional T_reg_ cells are identified in several autoimmune disorders including mutiple sclerosis (MS)^10^,^11^,^12^. One of the failures of T_reg_-cell-mediated immunoregulation is inadequate numbers of T_reg_ cells that may be due to defective induction of pT_reg_ cells in the periphery^13^. Thus, understanding of molecular mechanisms underlying pT_reg_-cell generation might provide deeper insights into physiological and pathological immune responses in autoimmune inflammatory diseases.

MicroRNAs (miRNAs) are single-stranded, small noncoding RNAs located in introns or exons of protein-coding genes as well as in non-coding genes^14^. miRNAs have been implicated in maintaining immune homeostasis during stress, such as inflammation, by regulating gene expression at post-transcriptional level^15^. Several studies have reported that specific miRNA signatures were observed for specialized T-cell subsets, and these miRNAs are dynamically regulated during T-cell maturation^16^,^17^. Dicer and Drosha are two essential components for the generation of miRNA, and loss of these factors leads to defects in lymphocyte differentiation and autoimmune inflammation^18^,^19^. Recently, accumulating evidence has demonstrated that miRNAs are also crucial for T_reg_-cell development, function and stability^17^,^18^,^19^,^20^. T_reg_ cells display a set of miRNAs that is distinct from conventional T cells^21^. However, intrinsic miRNAs involved in the polarization of T_reg_ cells from naive T cells *in vitro* and *in vivo* settings are largely undetermined.

In this study, we showed that miR-31 expression was triggered by TCR signalling, and downregulated by TGF-β1-induced FoxP3. The conditional deletion of miR-31 in CD4^+^ T cells led to enhanced induction of pT_reg_ cells in the periphery, and decreased severity of EAE. Retinoic acid (RA) regulates the expression of genes required for cell proliferation, differentiation and survival by binding its nuclear retinoic acid receptors (RARs) and retinoid X receptors (RXRs)^22^. Although RA has been shown to enforce pT_reg_-cell generation^23^, the mechanism by which RA promotes pT_reg_-cell induction is ill-defined. Unexpectedly, we here identified Gprc5a as a direct target of miR-31. Gprc5a is also known as retinoic acid-inducible protein 3 harbouring the functional RAR/RXR binding sites of RA in its core promoter^24^. Gprc5a was targeted by miR-31 through direct binding to its 3′-untranslated regions (3′-UTR), and its deficiency resulted in the impairment of pT_reg_-cell induction and increased EAE severity. Thus, our findings demonstrated that miR-31 negatively regulated pT_reg_-cell generation by targeting Gprc5a, suggesting a novel epigenetic mechanism for impaired pT_reg_-cell induction in autoimmunity.

## Results

### miR-31 expression is triggered by TCR signalling

**Figure 1:**
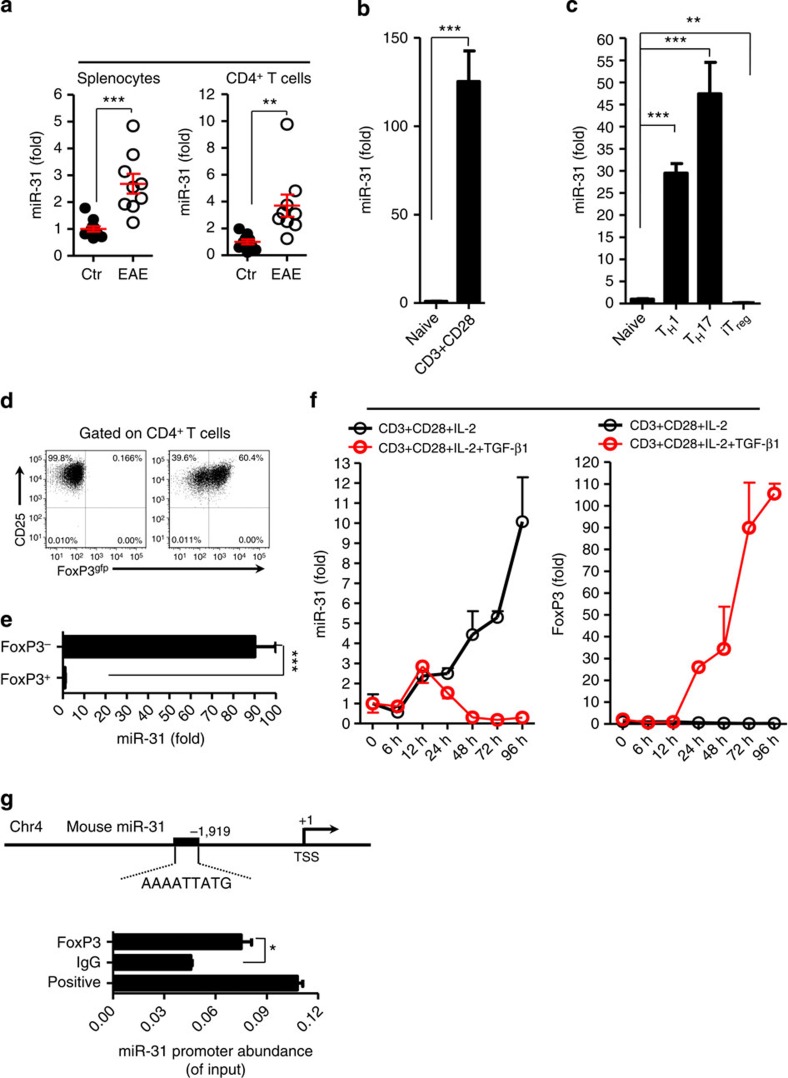
TCR signalling triggers expression of miR-31 that is downregulated by TGF-β1-induced FoxP3. (**a**) qPCR analysis of miR-31 expression in total splenocytes and sorted CD4^+^ T cells from healthy controls (Ctr) or EAE mice (*n*=9–11) 10 days post immunization. (**b**) miR-31 expression in naive T cells alone or cultured with anti-CD3, anti-CD28 mAb for 3 days (*n*=4). (**c**) qPCR analysis of miR-31 expression in naive T cells and polarized T_H_1, T_H_17 and iT_reg_ cells (*n*=4 per group); results are presented relative to miR-31 expression in isolated cells of control mice as in **a** or in naive T cells as in **b**,**c**. (**d**) Representative flow cytometry of FoxP3^gfp^ expression in naive T cells cultured with anti-CD3, anti-CD28 mAb and rmIL-2 in the absence (left panel, FoxP3^−^ T cells) or presence (right panel, FoxP3^+^ T cells) of TGF-β1 for 3 days. Numbers adjacent to outlined areas indicate per cent cells in each. (**e**) qPCR analysis of miR-31 expression in sorted FoxP3^−^ and FoxP3^+^ T cells (*n*=4 per group). (**f**) The time course of miR-31 and FoxP3 expression in naive T cells activated by anti-CD3, anti-CD28 mAb and rmIL-2 in the absence or presence of TGF-β1 for 96 h (*n*=3 per group; results presented as in **b**). (**g**) FoxP3 was immunoprecipitated from iT_reg_ cells. Immunoprecipitates were assayed for the expression levels of *miR-31* promoter. **P<0.01, ***P<0.001, two-tailed Student's *t*-test. Data are from one experiment representative of three (**a**–**e**) or two (**f**,**g**) independent experiments (mean±s.e.m.).

Report of FoxP3 mRNA harbouring the target sequence of miR-31 promoted us to investigate its role in the induction and/or function of T_reg_ cells which are vital for preventing autoimmune disease^21^. We induced EAE, an animal model of MS, with myelin oligodendrocyte glycoprotein peptide (MOG_35–55_) in mice to investigate expression pattern of miR-31 in pathogenic T cells in the tissue-specific autoimmune inflammation. miR-31 expression was assessed in splenocytes and sorted CD4^+^ T cells at day 10 post immunization. We found that the expression of miR-31 was significantly increased in both splenocytes and pathogenic CD4^+^ T cells in EAE mice compared with healthy controls (Fig. 1a). We next stimulated the TCR of naive T (CD4^+^CD25^−^CD62L^high^) cells with plate-coated anti-CD3- and soluble anti-CD28-specific antibodies, and we detected that the miR-31 expression was increased ∼125-fold in activated CD4^+^ T cells compared with untreated naive T cells (Fig. 1b). Together, these data suggest that TCR signalling induces miR-31 expression in CD4^+^ T cells.

Because the TCR signal coordinating with lineage-specific cytokines triggers naive T cells to differentiate into specialized effector cells, we sought to examine miR-31 expression in different T-cell subsets. We differentiated naive T cells *in vitro* under polarizing conditions for the generation of T_H_1, T_H_17 and iT_reg_ cells in cultures as these T-cell subsets are critical in the pathology of EAE^25^,^26^,^27^. At 4 days after activation, miR-31 expression was 29.5-fold higher in T_H_1 cells, 47.4-fold higher in T_H_17 cells, but there was 5.6-fold reduction in iT_reg_ cells than that of naive T cells (Fig. 1c), which suggested a possible regulatory role for miR-31 in CD4^+^ T-cell lineage differentiation. Because miR-31 has been implicated to negatively regulate FoxP3 expression in human T_reg_ cells^21^, we sought to investigate whether upregulation of miR-31 coincides with downregulation of FoxP3 during iT_reg_-cell induction. We polarized naive T cells derived from *FoxP3*^*gfp*^ reporter mice into iT_reg_ cells, and examined miR-31 expression in sorted CD25^+^FoxP3^−^ and CD25^+^FoxP3^+^ cells. The miR-31 expression in CD25^+^FoxP3^+^ cells was ∼90-fold lower than that in CD25^+^FoxP3^−^ population (Fig. 1d,e). These data demonstrate that miR-31 is preferentially diminished in iT_reg_ cells. Although the expression of miR-31 was slightly increased in tT_reg_ and pT_reg_ cells compared with iT_reg_ cells, its expression in either tT_reg_ or pT_reg_ was not significantly different between control and EAE mice, suggesting that T_reg_ cells maintain baseline miR-31 expression *in vivo* (Supplementary Fig. 1a). To further identify why iT_reg_ cells exhibit diminished levels of miR-31, we activated naive T cells with CD3- and CD28-specific antibodies in the absence or presence of TGF-β1, and measured the time-dependent appearance of miR-31. miR-31 abundance was gradually increased during the stimulation with CD3 and CD28 antibodies in the absence of TGF-β1, however, decreased at 12 h when FoxP3 was induced by adding TGF-β1 (Fig. 1f). Moreover, TGF-β1 dose-dependently decreased miR-31 expression in iT_reg_-cell differentiation (Supplementary Fig. 1b,c). Together, these data indicate that miR-31 expression might be downregulated by TGF-β1-induced FoxP3 during iT_reg_-cell induction *in vitro*. Database analysis revealed one potential FoxP3-binding site in the promoter element at −1919 upstream from the transcription start site (TSS) of mouse *miR-31* (Fig. 1g upper panel). To establish the possible binding of FoxP3 to the putative binding site in the promoter element of *miR-31*, we carried out chromatin immunoprecipitation (ChIP) assays. These assays showed a significant recruitment of FoxP3 to the putative *miR-31* promoter (Fig. 1g lower panel). Thus, our results suggest that FoxP3 possibly binds *miR-31* promoter and downregulates its expression during iT_reg_-cell *in vitro* differentiation.

### miR-31 conditional deletion ameliorates autoimmune disease

**Figure 2:**
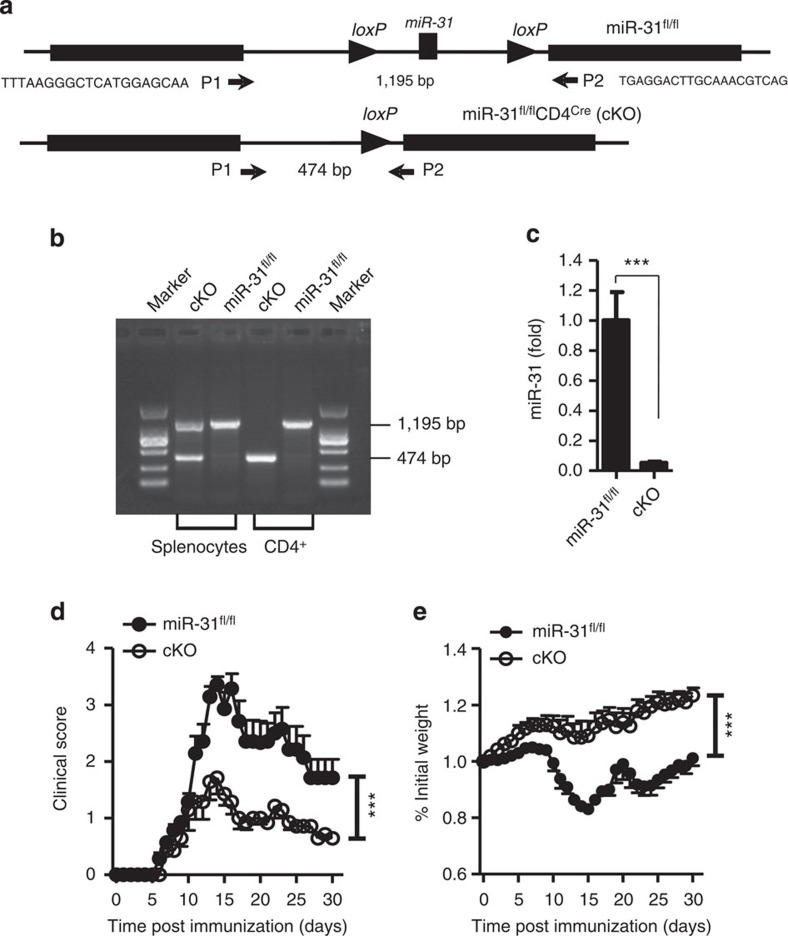
Alleviation of autoimmune disease in cKO mice. (**a**) Schematic representation of the *miR-31* locus and targeting strategy. Cre-mediated recombination of *loxP* sites in mice. Primers (P1 and P2) spanning the *loxP* sites were designed for genotyping floxed allele (1,195 bp) and deleted allele (474 bp). (**b**) PCR products of splenocytes and sorted CD4^+^ T cells derived from either *miR-31*^*fl/fl*^ control or *miR-31*^*fl/fl*^*CD4*^*Cre*^ (cKO) mice. (**c**) qPCR analysis to confirm the deletion of *miR-31* in CD4^+^ T cells derived from cKO mice. (**d**,**e**) Clinical scores and weight loss (mean±s.e.m.) of *miR-31*^*fl/fl*^ or cKO mice after the induction of EAE were assessed every day (*n*=7 per group). ***P<0.001, two-tailed Student's *t*-test for **c**, one-way analysis of variance for **d** and **e**. Data are representative of two (**c**) or three (**b**,**d** and **e**) independent experiments (mean±s.e.m.).

To determine whether miR-31 expressed by pathogenic CD4^+^ T cells is a functionally relevant regulator for the development of autoimmune inflammation, we used homologous recombination to generate mice with a *miR-31* allele flanked by *loxP* sites (floxed; Fig. 2a upper panel). The germline-transmitted mice were crossed with *CD4*^*Cre*^ transgenic mice to achieve a conditional knockout mouse model with a deleted *miR-31* allele in CD4^+^ T cells (Fig. 2a lower panel). To verify a specific deletion of *miR-31* in CD4^+^ T cells, we designed primers (P1 and P2) spanning the *loxP* sites (floxed allele, 1,195 bp; deleted allele, 474 bp) and genotyped mice using DNA of either splenocytes or sorted CD4^+^ T cells derived from *miR-31*^*fl/fl*^*CD4*^*Cre*^ (cKO) and *miR-31*^*fl/fl*^ control mice (Fig. 2a). We detected both floxed and deleted alleles in splenocytes of cKO mice, while only a floxed allele in splenocytes of *miR-31*^*fl/fl*^ control mice, a deleted allele in CD4^+^ T cells of cKO mice and a floxed allele in CD4^+^ T cells of *miR-31*^*fl/fl*^ control mice (Fig. 2b). Quantitative real-time PCR (qPCR) analysis confirmed a specific *miR-31* ablation in CD4^+^ T cells in cKO mice (Fig. 2c). These mice remained healthy without any detectable immune-mediated pathology at least for 32 weeks. By inducing EAE, we demonstrated that the specific miR-31 ablation in CD4^+^ T cells significantly decreased its severity accompanied by an evident prevention of weight loss in cKO mice compared with *miR-31*^*fl/fl*^ controls (Fig. 2d,e). Moreover, the deletion of miR-31 led to a marked decrease in infiltration of inflammatory cells and demyelination in spinal cord of cKO mice with EAE (Supplementary Fig. 2a–c). Thus, using genetic approach, we clearly demonstrated the significant impact of miR-31 expressed by CD4^+^ T cells on the development of autoimmunity.

### miR-31 skews the CD4 T-cell-mediated immune balance

**Figure 3:**
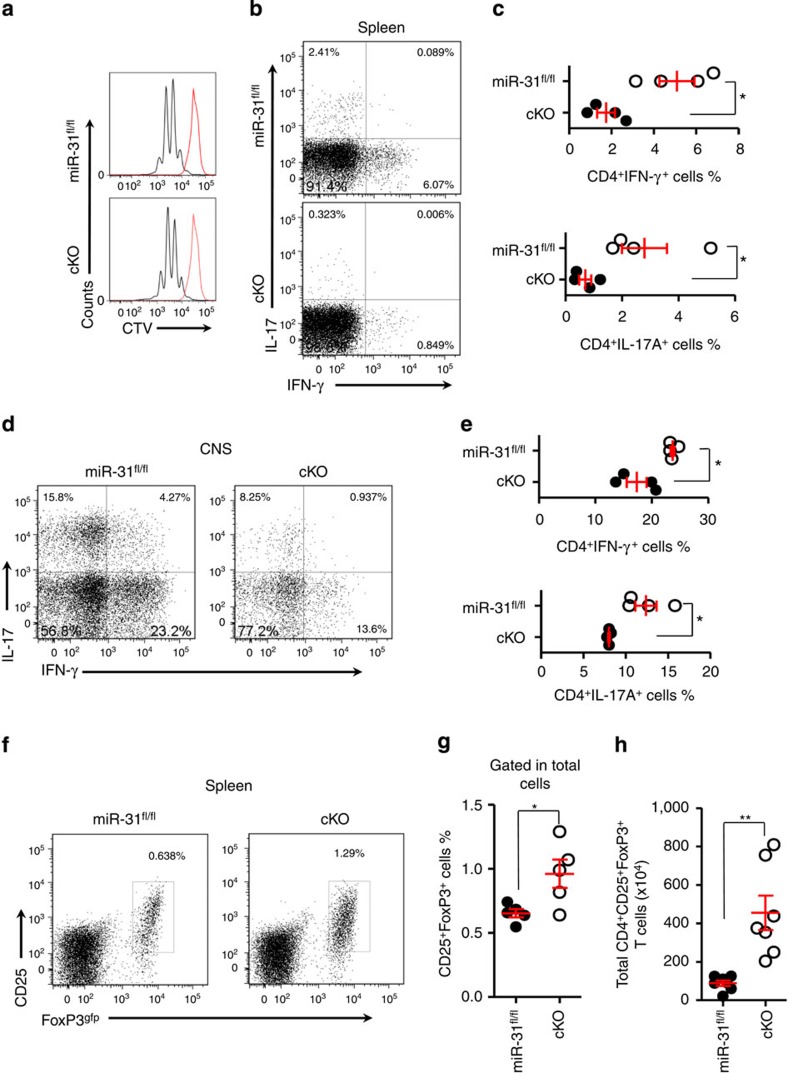
miR-31 contributes to altered balance between pathogenic T_H_1/T_H_17 cells and T_reg_ cells in autoimmunity. (**a**) Representative flow cytometric analysis of CTV fluorescence dilution of *miR-31*^*fl/fl*^ or cKO naive T cells cultured with anti-CD3, anti-CD28 mAb and rmIL-2 for 3 days. Red lines indicate non-stimulated controls. (**b**,**c**) Flow cytometry of T_H_1 and T_H_17 cells in inflamed spleen of *miR-31*^*fl/fl*^ and cKO mice 14 days after the induction of EAE. (**d**,**e**) Flow cytometry of T_H_1 and T_H_17 cells in CNS of *miR-31*^*fl/fl*^ and cKO mice 14 days after the induction of EAE. (**f**,**g**) Flow cytometric analysis of T_reg_ cells in the spleen of *miR-31*^*fl/fl*^*/FoxP3*^*gfp*^ and cKO/*FoxP3*^*gfp*^ mice 14 days after the induction of EAE (*n*=5, gated on total cells). Numbers in quadrants or adjacent to outlined areas indicate per cent cells in each. (**h**) Absolute numbers of T_reg_ cells in the spleen of *miR-31*^*fl/fl*^*/FoxP3*^*gfp*^ and cKO/*FoxP3*^*gfp*^ mice 14 days after the induction of EAE (*n*=7). *P<0.05, **P<0.01, two-tailed Student's *t*-test. Data are from one experiment representative of at least two independent experiments (mean±s.e.m.).

To assess how deletion of miR-31 in CD4^+^ T cells reduced the severity of progressive EAE, we analysed T-cell frequency and activation in non-immunized cKO mice. By flow cytometric analysis, we observed no substantial changes in T-cell numbers and activation status in the thymus in cKO mice compared with *miR-31*^fl/fl^ controls (Supplementary Fig. 3a,b). T-cell proliferation in response to stimulation via TCR-CD28 was also similar in *miR-31*^fl/fl^ and cKO T cells as determined by CellTrace Violet (CTV) fluorescence (Fig. 3a). These data suggest that miR-31 is dispensable for T-cell development, activation and proliferation. We next analysed T_reg_-cell frequency in non-immunized mice, and found that miR-31 deficiency did not change the proportion of T_reg_ cells in the thymus and periphery, indicating that miR-31 had no impact on tT_reg_ and pT_reg_-cell development (Supplementary Fig. 4a–c). T_H_1 and T_H_17 cells are inflammatory cells that develop during tissue-specific inflammatory responses and play a critical role in enhancing tissue inflammation^25^,^27^. Therefore, we investigated inflamed spleen and central nervous system (CNS) from *miR-31*^fl/fl^ and cKO mice for the presence of IFN-γ-producing (T_H_1) and IL-17-producing (T_H_17) CD4^+^ T cells during EAE. In contrast to *miR-31*^fl/fl^ controls, cKO mice showed a significant reduction of T_H_1 and T_H_17 cell proportion not only in inflamed spleen (Fig. 3b,c) but also in CNS (Fig. 3d,e) 14 days post immunization with MOG_35–55_. These data suggest that the development of inflammatory T_H_1 and T_H_17 cells in cKO mice is impaired during the induction phase of autoimmune disease. We next investigated whether the decreased encephalitogenic potential of CD4^+^ T cells in cKO mice was a consequence of increased peripheral T_reg_-cell generation during EAE. On day 14 post immunization, we observed a marked increase in the proportion and absolute numbers of T_reg_ cells in the periphery of cKO mice (Fig. 3f–h). Moreover, there was no significant difference of tT_reg_ frequency in *miR-31*^fl/fl^ and cKO mice with EAE (Supplementary Fig. 4d). Together, these results demonstrate that miR-31 skews the balance between pathogenic T_H_1/T_H_17 cells and T_reg_ cells in the periphery during autoimmune inflammation.

### miR-31 limits pT_reg_-cell induction

**Figure 4:**
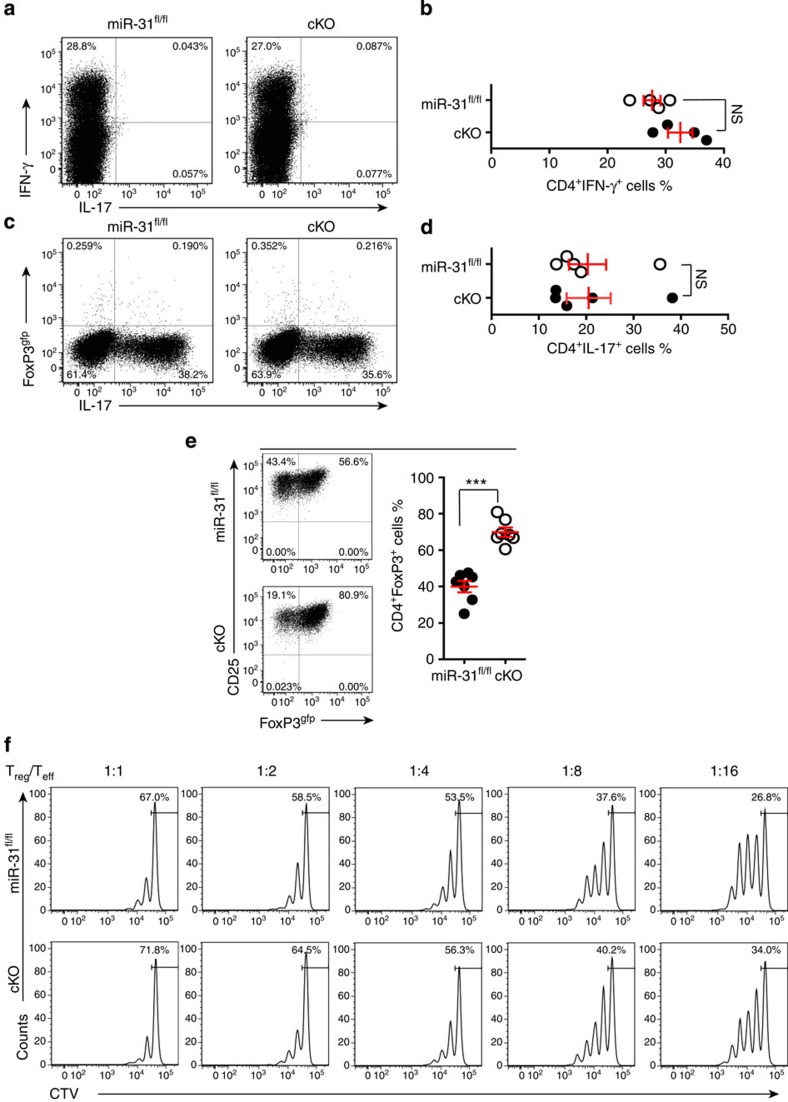
miR-31 restrains iT_reg_ cell differentiation *in vitro.* *FoxP3*^*gfp*^ reporter mice were crossed with either *miR-31*^*fl/fl*^ or cKO mice. (**a**–**d**) Flow cytometry of T_H_1 and T_H_17 cells polarized from naive T cells of *miR-31*^*fl/fl*^*/FoxP3*^*gfp*^ and cKO/*FoxP3*^*gfp*^ mice (*n*=4–5). Numbers in quadrants indicate per cent cells in each. (**e**) Flow cytometric analysis of *in vitro*-induced iT_reg_ cells from naive T cells of *miR-31*^*fl/fl*^*/FoxP3*^*gfp*^ and cKO/*FoxP3*^*gfp*^ mice (*n*=7). Numbers adjacent to outlined areas indicate per cent cells in each. (**f**) FoxP3^gfp+^ T_reg_ and naive T cells (T_eff_) were sorted by flow cytometry. The CTV-labelled T cells (1 × 10^5^) were cultured in 96-well plates for 72 h together with a decreasing ratio of sorted *miR-31*^*fl/fl*^ or cKO T_reg_ cells in the presence of anti-CD3 (1 μg ml^−1^) plus γ-irradiated antigen-presenting cells (1 × 10^5^). The suppressive function of T_reg_ cells was determined by the proliferation of activated T_eff_ cells on the basis of CTV dilution. NS, not significant, ***P<0.001, two-tailed Student's *t*-test. Data are from one experiment representative of at least two independent experiments (mean±s.e.m.).

Because miR-31 exhibited a distinct expression pattern in differentiated CD4^+^ T-cell subsets, we postulated that the intrinsic miR-31 may regulate their *in vitro* generation. We sorted naive T cells from *miR-31*^*fl/fl*^ and cKO mice, and polarized them into T_H_1, T_H_17 and iT_reg_ cells under lineage-specific conditions *in vitro*. After 4 days culture, we found no significant change for the differentiation of T_H_1 and T_H_17 cells from naive T cells of cKO mice compared with *miR-31*^*fl/fl*^ control mice (Fig. 4a–d). Of note, the lack of miR-31 markedly induced iT_reg_-cell differentiation in culture (Fig. 4e). Thus, these data suggest that miR-31 deficiency preferentially enhanced the generation of TGF-β1-induced iT_reg_ cells *in vitro*. Helios is potentially a marker, which could distinguish tT_reg_ cells from pT_reg_ cells^28^. We injected intravenously bone marrow cells (5 × 10^6^) from either *miR-31*^fl/fl^ or cKO mice into lethally irradiated C57BL/6J recipient mice to generate bone marrow chimeric mice. Eight weeks after bone marrow transplantation, EAE was induced in all chimeric mice. On day 14 post immunization, we analysed the frequency of Helios^−^FoxP3^+^ pT_reg_ cells and Helios^+^FoxP3^+^ tT_reg_ cells in the spleen of chimeric mice by flow cytometry. By Helios staining, we demonstrated that the conditional deletion of miR-31 led to increased numbers of Helios^−^ pT_reg_ cells, whereas Helios^+^ tT_reg_ cells had no significant change *in vivo* (Supplementary Fig. 5). Together, our data indicate that miR-31 limits pT_reg_-cell induction in autoimmunity. We next examined the suppressive capacity of *miR-31*^*fl/fl*^ and cKO T_reg_ cells. CTV dilution determined that cKO T_reg_ cells inhibited T-cell proliferation to the same extent as *miR-31*^*fl/fl*^ T_reg_ cells (Fig. 4f). pT_reg_ cells had robust suppression and enhanced stability, suppressed ongoing EAE^29^. Given the fact that pT_reg_ cells converted from conventional T cells play a critical role in the control of development of EAE or other autoimmune diseases^8^,^26^,^30^,^31^,^32^, we here provided strong evidence that promoting generation of pT_reg_ cells by disrupting miR-31 was likely responsible for the observed phenotype.

### Gprc5a is a target of miR-31

**Figure 5:**
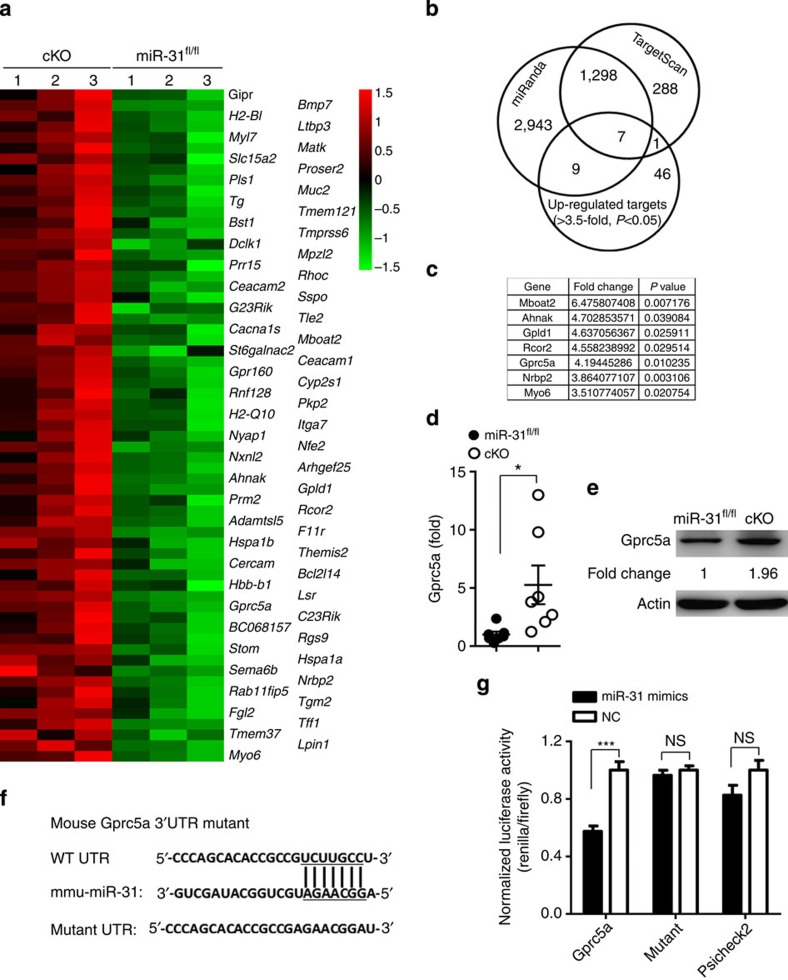
Gprc5a is directly targeted by miR-31. (**a**) Total RNAs of polarized iT_reg_ cells derived from 3 *miR-31*^*fl/fl*^ controls and 3 cKO mice were used for a microarray analysis (41,174 genes in total). Transcripts of top 63 genes were found to be upregulated in iT_reg_ cells of cKO mice. (**b**,**c**) TargetScan and miRnada predicted 1,305 potential targets of miR-31. The overlapping seven genes were defined as ACCEPT genes. (**d**,**e**) qPCR or western blot analysis of Gprc5a expression in polarized iT_reg_ cells derived from *miR-31*^*fl/fl*^ and cKO mice. (**f**) WT and point-mutated 3′-UTR reporter constructs. (**g**) Luciferase activity was determined in NIH3T3 cells that were transfected with miR-31 mimics and the indicated 3′-UTR reporter construct or with the indicated WT or point-mutated 3′-UTR reporter construct (WT UTR or mutant UTR). Results (**d**) are presented as the ratio of mRNA to the β-actin, relative to that in controls. *P<0.05; ***P<0.001, NS, not significant, two-tailed Student's *t*-test. Data (**d**,**e** and **g**) are representative of at least two independent experiments (mean±s.e.m.).

To elucidate the mechanisms, we combined microarray gene expression analysis and target prediction to look for putative targets of miR-31. Using a combination of these two approaches, we identified seven predicted target genes that were upregulated in polarized iT_reg_ cells derived from cKO mice (Fig. 5a–c). To confirm accuracy of the microarray data, we validated these potential target genes by increasing sample numbers. We found one predicted target of miR-31, Gprc5a, was significantly upregulated at mRNA levels, and increased by more than 5.0-fold in cKO iT_reg_ cells compared with *miR-31*^*fl/fl*^ controls (Supplementary Fig. 6 and Fig. 5d). In contrast to *miR-31*^*fl/fl*^ iT_reg_ cells, Gprc5a protein level was also increased by 1.96-fold in cKO iT_reg_ cells (Fig. 5e). Gprc5a was reported to be regulated directly by RA via its receptors, RARs and RXRs^33^,^34^. These nuclear RA receptors bind to the *Gprc5a* promoter for its transcriptional activity^24^. We next generated a reporter construct that includes the 3′-UTR of Gprc5a mRNA. In contrast to a control construct lacking the target sequence, miR-31 overexpression led to significantly decreased luciferase activity derived from the construct expressing the target sequence (Fig. 5f,g). Thus, our data demonstrate that miR-31 is capable of directly targeting a sequence within the 3′-UTR of Gprc5a mRNA and that Gprc5a is one of the key targets of miR-31 in T_reg_-cell differentiation.

### Gprc5a is critical for pT_reg_-cell differentiation

**Figure 6:**
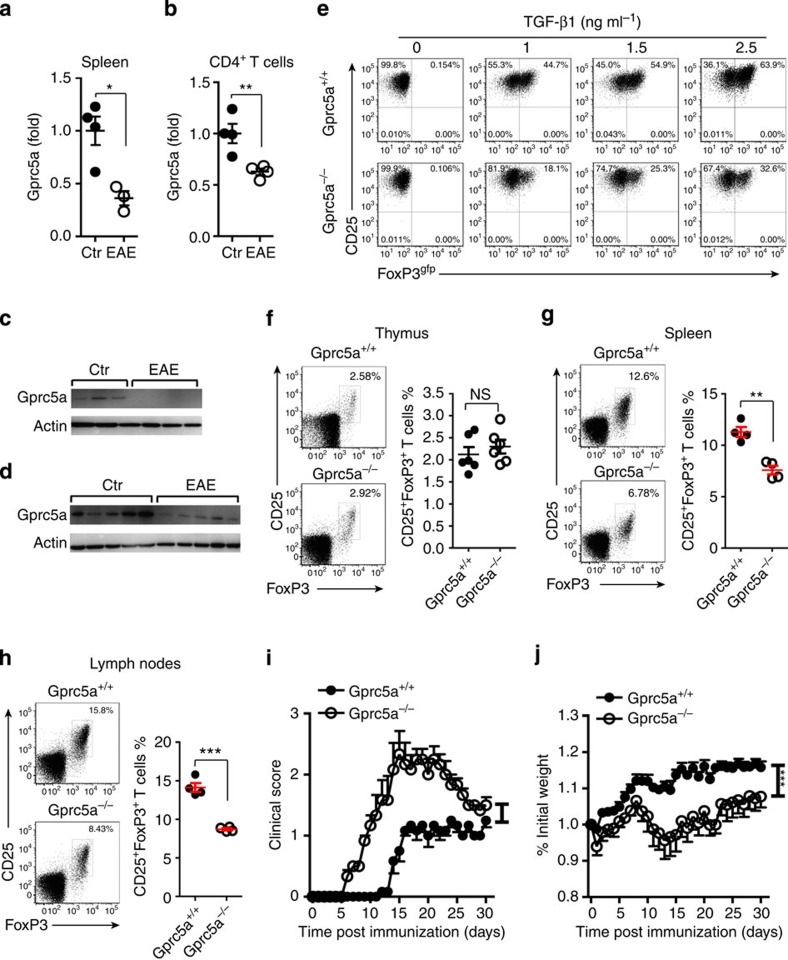
Gprc5a deficiency decreases pT_reg_-cell differentiation and promotes autoimmune inflammation. (**a**,**b**) Splenocytes were prepared from unimmunized control mice (Ctr) or EAE mice 10 days after immunization (*n*=3–4). qPCR analysis of Gprc5a expression in total splenocytes or in sorted CD4^+^ T cells. (**c**,**d**) Western blot analysis of Gprc5a in splenocytes and CNS infiltrating cells derived from healthy controls (Ctr) or EAE mice. (**e**) Naive T cells were sorted from *Gprc5a*^*+/+*^ and *Gprc5a*^−*/*−^ mice. Flow cytometry of polarized iT_reg_ cells in the presence of different concentrations of TGF-β1. (**f**) Flow cytometric analysis of T_reg_ cells in thymus from 6-week-old *Gprc5a*^*+/+*^ and *Gprc5a*^−*/*−^ mice (gated on CD4^+^ T cells). (**g**,**h**) Flow cytometric analysis of T_reg_ cells in the inflamed spleen and lymph nodes *Gprc5a*^*+/+*^ and *Gprc5a*^−*/*−^ mice 14 days after the induction of EAE (gated on CD4^+^ T cells). Numbers adjacent to outlined areas indicate per cent cells in each. (**i**,**j**) Clinical scores and weight loss (mean±s.e.m.) of *Gprc5a*^*+/+*^ or *Gprc5a*^−*/*−^ mice after the induction of EAE were assessed every day (*n*=7). *P<0.05, **P<0.01, ***P<0.001, NS, not significant, two-tailed Student's *t*-test for **a**,**b**,**f**,**g** and **h**; one-way analysis of variance for **i**,**j**. Data are representative of at least two independent experiments (mean±s.e.m.).

Gprc5a was reported to be expressed preferentially in lung tissue and to be a putative lung tumour suppressor gene^35^. The functional analysis of Gprc5a in T-cell differentiation and autoimmunity is not yet performed. We induced EAE, and measured Gprc5a expression in inflamed spleen and sorted CD4^+^ T cells. In contrast with non-immunized controls, Gprc5a expression was significantly decreased in spleen and CD4^+^ T cells in EAE mice, and this might be the consequence of increased miR-31 under inflammatory conditions (Fig. 6a,b). Western blot analysis confirmed that expression of Gprc5a protein was much lower in spleen and CNS of EAE mice than those of healthy controls (Fig. 6c,d). We assessed the role of Gprc5a in pT_reg_-cell generation using *Gprc5a*^−/−^ mice^35^. Gprc5a deficiency resulted in a marked decrease in the TGF-β1-mediated induction of iT_reg_ cells, but had no impact on the induction of T_H_1 and T_H_17 cells (Fig. 6e and Supplementary Fig. 7a,b), suggesting that Gprc5a preferentially regulates FoxP3 expression. Thus, our data demonstrate that Gprc5a is a novel regulator in iT_reg_-cell generation. Interestingly, consistent with the observation in *miR-31* cKO mice, we found that Gprc5a deficiency did not affect tT_reg_-cell generation in healthy mice (Fig. 6f). To test the impact of Gprc5a on the T_reg_-cell response during inflammation, we analysed the frequency of T_reg_ cells in inflamed spleen in *Gprc5a*^−/−^ mice after the induction of EAE. Gprc5a deficiency resulted in a significant decrease in the percentage of pT_reg_ cells compared with WT controls (Fig. 6g, h), suggesting that Gprc5a is critically required for pT_reg_-cell generation *in vivo in* autoimmune disease. Notably, *Gprc5a*^−/−^ mice developed EAE not only much earlier, but also more severe than *Gprc5a*^+/+^ mice (Fig. 6i). Moreover, an excessive weight loss was displayed in *Gprc5a*^−/−^ mice compared with *Gprc5a*^+/+^ mice (Fig. 6j). To further investigate whether miR-31 affects EAE development via regulating Gprc5a *in vivo*, we generated double knockout (DKO) mice by crossing *miR-31* cKO mice with *Gprc5a*^−*/*−^ mice. We demonstrated that the severity of EAE was significantly reduced in cKO mice compared with *Gprc5a*^−*/*−^ mice, however, the disease phenotype was completely restored when *Gprc5a* was deleted in the cKO mice (Supplementary Fig. 7c). Collectively, our observations indicate that Gprc5a is regulated by miR-31, and functionally involved in the development of EAE. The beneficial effect of RA is possibly due to its stimulation of Gprc5a expression to promote pT_reg_-cell generation in tissue-specific autoimmune inflammation.

## Discussion

T_reg_ cells have been reported to be capable of controlling CNS autoimmunity in several CD4^+^ T-cell-driven EAE models. T_reg_-cell frequency within the CNS was increased during the recovery phase of actively induced EAE^36^,^37^, and the adoptive transfer of T_reg_-cells ameliorated EAE symptoms^36^,^38^. Furthermore, depletion of T_reg_ cells with anti-CD25 mAb has been demonstrated to exacerbate EAE^36^. More importantly, reduced T_reg_-cell proliferative potential and cloning frequency were identified in patients with MS^12^,^39^. Thus, regulators of T_reg_-cell generation are considered to harbour valuable potential for clinical applications in the treatment of autoimmune disorders. Here, we report that miR-31 expression in CD4^+^ T cells was triggered by TCR signalling, and downregulated by TGF-β1-mediated FoxP3. Its conditional deletion substantially enhanced the pT_reg_-cell induction and ameliorated disease severity in the EAE model. Mechanistically, we have proven that by targeting Gprc5a, a known retinoic acid-inducible protein, miR-31 promoted the generation of pT_reg_ cells *in vivo*. Gprc5a is a functional target of miR-31, and its deficiency resulted in impaired pT_reg_-cell generation and increased EAE severity.

Antigen-specific stimuli delivered through the TCR cooperates with antigen-nonspecific cytokines to support proliferation and differentiation of distinct T_H_ cell subsets^40^. However, it has become increasingly clear that miRNAs, post-transcriptional regulators, are involved in driving T_H_ cell differentiation and lineage commitment^41^. A selective effect of miR-31 on pT_reg_-cell differentiation could be explained by the differential requirement of TCR signalling in the induction of these T-cell lineages. A low antigen dose of a high-affinity TCR ligand favours to induce pT_reg_ cells *in vivo*^9^, whereas high doses of TCR stimulation prevents FoxP3 induction and pT_reg_-cell generation through activating NF-κB signalling^42^. Indeed, we have determined that the activation of NF-κB induced miR-31 expression through a direct binding of p65 to its promoter (to be published elsewhere). Thus, it is possible that in the absence of TGF-β1, TCR stimulation at high doses elicits activation of NF-κB, which directly triggers the expression of miR-31 inhibiting FoxP3 levels in CD4^+^ T cells. However, TCR stimulation at low doses induces FoxP3, which may downregulate miR-31 expression through binding to its promoter, providing a feedback loop during pT_reg_-cell differentiation.

Several miRNAs have been reported to impact T_reg_-cell development and function. miR-155 is highly expressed in T_reg_ cells, facilitates T_reg_-cell homeostasis by repressing *Socs1* and its deficiency results in decreased numbers of both tT_reg_ cells and pT_reg_ cells^43^. miR-21 indirectly acts as a positive regulator of human FoxP3 expression^21^. miR-146a is critical for T_reg_-cell-mediated control of T_H_1 responses via targeting Stat1 (ref. 44). Despite that miR-17∼92 is dispensable for the development of tT_reg_ cells *in vivo*, miR-17∼92 ablation reduces the frequency of MOG_35–55_-specific pT_reg_ cells during EAE^45^. miR-10a is induced by TGF-β1 and RA, and promotes the differentiation of pT_reg_ cells through inhibiting Bcl-6 (ref. 46). Collectively, these T_reg_-cell-associated miRNAs are all enriched in T_reg_ cells compared with conventional T cells, and function as positive regulators. Our data demonstrated that miR-31 was preferentially diminished in T_reg_ cells, was downregulated by FoxP3, and negatively regulated naive CD4^+^ T-cell differentiation into pT_reg_ cells. The conditional deletion of *miR-31* in CD4^+^ T cells resulted in enhanced induction of pT_reg_ cells in the periphery, and decreased severity of autoimmune disease. Thus, we highlight miR-31 acts as a negative regulator for pT_reg_-cell generation *in vivo*. Although different targets of miR-31 were identified, its similar effect was also reported previously for human T_reg_ cells^21^. Our findings are inconsistent with a recent report which demonstrated that growth factor independent 1 (Gfi-1) was underexpressed in pT_reg_ cells, downregulated by TGF-β1 and limited the pT_reg_-cell differentiation^47^.

miR-31 is the only member of a broadly conserved miRNA ‘seed family' that is present in vertebrates and Drosophila^48^. miR-31 regulates keratinocyte differentiation through inhibiting hypoxia-inducible factor 1 (ref. 49). Furthermore, in contrast to other T-cell subsets, miR-31 has been shown to be downregulated in human T_reg_ cells^21^. This raises the intriguing possibility that miR-31 may be preferentially diminished in T_reg_ cells and its upregulation in CD4^+^ T cells under inflammatory stress may limit pT_reg_ cell induction in human autoimmune diseases.

RA has been proven to facilitate pT_reg_-cell generation^23^. RA regulates Gprc5a transcriptional activity by binding to its receptors, RARs and RXRs^24^,^33^,^34^. So far, the role of Gprc5a in the T-cell differentiation programme is not investigated. We here showed that Gprc5a deficiency led to a severe defect in *in vitro*- and *in vivo*-generation of T_reg_ cells, as well as increased severity of inflammatory CNS phenotypes, indicating that this may be one of the mechanisms by which RA inhibits autoimmune reactions *in vivo*. However, the molecular mechanism by which Gprc5a promotes T_reg_-cell generation and suppresses autoimmune disease is subjected to further investigation. Nevertheless, by generating *miR-31* and its target *Gprc5a* DKO mice we clearly show that miR-31 promotes the development of autoimmune disease through inhibiting Gprc5a.

In summary, our results demonstrate that miR-31 inhibits pT_reg_-cell generation through directly targeting Gprc5a, a retinoic acid-inducible protein and promotes autoimmunity, therefore, providing the first *in vivo* genetic evidence that miR-31 and its novel target Gprc5a are critical intrinsic factors for controlling physiological and pathological immune responses regulated by pT_reg_ cells.

## Methods

### Mice

C57BL/6J mice (stock number: 000664), B6.Cg-*FoxP3*^tm2Tch/J^ mice (stock number: 006772, designated as *FoxP3*^*gfp*^) and *CD4*^*Cre*^ mice (stock number: 017336) were purchased from The Jackson Laboratory (Bar Harbor, ME). B6.129S6-Rag2^tm1Fwa^ N12 (*RAG2*^−*/*−^) mice were purchased from Taconic Labs (Hudson, NY). *Gprc5a*^−*/*−^ mice were generated as previously reported^35^. Mice were kept under specific pathogen-free conditions in compliance with the National Institutes of Health *Guide for the Care and Use of Laboratory Animals* with the approval (SYXK-2003-0026) of the Scientific Investigation Board of Shanghai Jiao Tong University School of Medicine, Shanghai, China. To ameliorate any suffering of mice observed throughout these experimental studies, mice were euthanized by CO_2_ inhalation.

### Generation of *miR-31* ^*fl/fl*^ and *miR-31* ^*fl/fl*^ *CD4* ^*Cre*^ mice

The *miR-31* locus (mmu-mir31 ENSMUSG00000065408, http://www.ensembl.org/index.html) is on the chromosome 4 (Mus musculus) and encodes the *miR-31*. To create *loxP-miR-31-loxP* mice, a targeting vector was designed to insert with an frt-flanked PGK-neo cassette and a loxP site upstream of *miR-31*, and a second loxP site downstream. LoxP site is a 34 bp length DNA sequence that can be recognized by Cre recombinase catalyses. If two loxP sites are introduced in the same orientation into a genomic locus, expression of Cre results in the deletion of the loxP-flanked DNA sequence. After linearization, the vector was electroporated into 129S6-derived embryonic stem (ES) cells. The collected ES cells were screened with 300 μg ml^−1^ G418 and 2 μM Gan C for 8 days and ascertained by PCR. The ES cells with right homologous recombination were injected into blastocyst. After birth, the chimeric mice were bred with 129S mice to generate the heterozygotes. At this point, mutant mice were bred with Flp recombinase-expressing mice to remove the frt-flanked neo cassette. The resulting *loxP-miR-31-loxP* mice were backcrossed into C57BL/6J background for eight generations and bred with *CD4*^*Cre*^ transgenic mice. P1 and P2 were used to genotype the *miR-31* floxed allele (1,195 bp) and the *miR-31* deleted allele (474 bp). P1, 5′- TTTAAGGGCTCATGGAGCAA -3′; P2, 5′- TGAGGACTTGCAAACGTCAG -3′. Excision by *CD4*^*Cre*^ was complete for all pups used in experiments. In some experiments, these mice were further crossed with *FoxP3*^*gfp*^ mice to generate mice that express green fluorescent protein (GFP) in their T_reg_ cells.

### Induction of EAE

EAE was induced by complete Freund's adjuvant (CFA)-MOG_35–55_ peptide immunization (China Peptides Biotechnology) and scored daily. Briefly, C57BL/6J mice were injected subcutaneously into the base of the tail with a volume of 200 μl containing 300 μg MOG_35–55_ peptide emulsified in CFA (Sigma-Aldrich). Mice were also injected intravenously with 200 ng of pertussis toxin (Merck-Calbiochem) on day 0 and 2 post immunization. All the reagents used for *in vivo* experiments were free of endotoxin. Mice were monitored daily for the development of disease which was scored according to the following scale: 0, no symptoms; 0.5, partially limp tail; 1, completely limp tail; 1.5, impaired righting reflex; 2, hind limb paresis; 2.5, hind-limb paralysis; 3, forelimb weakness; 4, complete paralysis; 5, moribund or death.

### T-cell isolation and sorting

Peripheral T cells were obtained from the spleen and lymph nodes of 6-week-old mice. Naive CD4^+^ T cells (CD4^+^CD25^−^CD62L^high^) were sorted by FACSAria III (BD Biosciences) after enrichment of CD4^+^ T cells by the mouse CD4^+^ T cell Isolation Kit (Miltenyi). For the isolation of naive CD4^+^ T cells and nT_reg_ cells from *Foxp3*^*gfp*^ mouse, CD4^+^CD25^−^GFP^−^CD62L^high^ cells and CD4^+^CD25^+^GFP^+^ cells were isolated, respectively. Cell purity was >94% as determined by flow cytometry. CNS-infiltrating mononuclear cells from EAE mice were prepared by Percoll (GE Healthcare) gradient separation.

### *In vitro* T_reg_-cell differentiation

All the cultures of T cells used RPMI-1640 medium (Gibco) supplemented with 10% heat-inactivated fetal bovine serum (Gibco), 2 mM L-glutamine (Gibco), 100 IU ml^−1^ penicillin, 100 μg ml^−1^ streptomycin, 10 mM HEPES (Gibco) and 5 mM β-mercaptoethanol (Gibco). The naive CD4^+^CD25^−^Foxp3^gfp-^CD62L^hi^ T cells were activated with plate-bound anti-CD3 (5 μg ml^−1^; 145-2C11; BD Biosciences) plus soluble anti-CD28 (2 μg ml^−1^; 37.51; BD Biosciences). T_H_1-cell differentiation conditions included 10 ng ml^−1^ rmIL-12 (R&D Systems) and 10 μg ml^−1^ anti-IL-4 (11B11; Biolegend). The T_H_17 cell differentiation conditions included 20 ng ml^−1^ rmIL-6 (R&D Systems), 3 ng ml^−1^ rmTGF-β1 (R&D Systems), 10 μg ml^−1^ anti-IL-4 (11B11; Biolegend) and 10 μg ml^−1^ anti-IFN-γ (XMG1.2; eBioscience). The iT_reg_-cell differentiation conditions included 5 ng ml^−1^ rmTGF-β1 (R&D Systems) and 10 ng ml^−1^ rmIL-2 (R&D Systems).

### Flow cytometry

Cytokines, transcriptional factors and surface markers were evaluated by flow cytometry with a FACSCanto II (BD Biosciences). To detect intracellular expression of IL-17A, IFN-γ in CD4^+^ T cells, lymph nodes or CNS (purified with Percoll) were first treated with 750 ng ml^−1^ ionomycin (Sigma), 50 ng ml^−1^ phorbol 12-myristate 13-acetate (PMA) (Sigma) and GolgiPlug (BD Biosciences) for 4–6 h at 37 °C. Cells were fixed and permeabilized with the Foxp3 Staining Buffer Set (eBioscience) or BD Cytofix/Cytoperm (BD Biosciences) and were stained with fluorescent antibodies. After washing, stained cells were assayed with a BD Biosciences FACSCanto II flow cytometer and data were analysed with FlowJo software. For flow cytometry, monoclonal antibodies against CD4 (clone GK1.5), CD8 (clone 53-6.7), CD62L (clone MEL-14), CD44 (clone IM7), CD25 (clone PC61.5), IL-17A (clone eBio17B7), IFN-γ (clone XMG1.2), Helios (22F6, Biolegend) and FoxP3 (clone FJK-16s) were from eBioscience and CD3 (clone 145-2C11) was from Biolegend.

### RNA reverse transcription and real-time quantitative PCR

Total RNA was isolated using Trizol (Invitrogen) according to the manufacturer's instructions. RNA was quantified spectrophotometrically, and 1 μg of total RNA was reverse transcribed into cDNA using SuperScript III (Invitrogen, Carlsbad, CA) in the presence of random hexamers and oligo dT primers (Invitrogen). The cDNA samples were distributed on plates at 200 ng per well and run in triplicate. qPCR was carried out with the FastStart Universal SYBR Green Master (Roche) in a ABI 7500 Fast Real-Time PCR system or ViiA 7 Real-Time PCR System (Applied Biosystems). Primer sequences were listed in Supplementary Table 1. To measure mature miR-31 levels, 50 ng of total RNA was reverse-transcribed using the TaqMan miRNA reverse transcription kit, miR-31 RT primers and U6 snRNA (Applied Biosystems). The cDNAs were then analysed by qPCR using the TaqMan probes for miR-31 and U6 snRNA (Applied Biosystems). Quantification of relative miRNA expression was measured by the comparative CT (critical threshold) method, normalized to endogenous U6 expression and determined by the formula 2^−ΔΔCT^.

### Chromatin immunoprecipitation assay

ChIP assays were performed using the SimpleChIP enzymatic chromatin immunoprecipitation kit (Cell Signaling Technology) according to the manufacturer's protocol with minor modifications. In brief, the cells were collected and crosslinked with 1% (v/v) formaldehyde for 10 min at room temperature. Subsequently, nuclei were isolated by the lysis of cytoplasmic fraction and chromatin was digested into fragments of 150–900 bp by micrococcal nuclease (400 gel units) for 20 min at 37 °C, followed by ultrasonic disruption of the nuclear membrane using a standard microtip and a Branson W250D Sonifier (four pulses, 60% amplitude, duty cycle 40%). The sonicated nuclear fractions were divided for input control and for overnight incubated at 4 °C with 5 μg either anti-FoxP3 Ab (FJK-16 s; eBioscience) or the negative control IgG (Cell Signaling Technology). After incubation with 30 μl of ChIP grade protein G-agarose beads for 2 h at 4 °C, the antibody-protein–DNA complexes were then eluted from the beads and digested by Proteinase K (40 μg) for 2 h at 65 °C, followed by spin column-based purification of the DNA. Finally, genomic DNA recovered from the ChIP assays were qPCR amplified with primers specific to the FoxP3-binding elements of the *miR-31* promoter region. The primers used for detection of *miR-31* promoter sequences were listed (Supplementary Table 1). The specificity of each primer set was verified by analysing the dissociation curve of each gene-specific PCR product.

### *In vitro* T_reg_-cell suppression assay

T_reg_ cells were isolated from the spleen of mice by sorting with flow cytometry based on cell surface markers (CD4, CD25 and FoxP3^gfp^). Naive CD4^+^ T cells were isolated from the spleen of mice by sorting with flow cytometry based on cell surface markers (CD4^+^, CD25^−^, CD62L^hi^ and FoxP3^gfp^). Splenocytes from *RAG2*^−*/*−^ mice lacking mature T and B lymphocytes were used as antigen presenting cells. The purified naive CD4^+^ T cells were labelled for 15 min at 37 °C with 10 μM CTV (Life Technologies) and the CTV-labelled T cells (1 × 10^5^) were cultured in 96-well plates for 72 h together with an increasing ratio of sorted T_reg_ cells in the presence of anti-CD3 (1 μg ml^−1^) plus γ-irradiated antigen-presenting cells (1 × 10^5^). The suppressive function of T_reg_ cells was determined by measurement of the proliferation of activated CD4^+^ effector T cells on the basis of CTV dilution.

### Generation of bone marrow chimeric mice

Bone marrow cells were flushed from *miR-31*^fl/fl^ or cKO donor mice, and 5 × 10^6^ T-cell-depleted bone marrow cells were transplanted into each C57BL/6J host mouse with total-body irradiation of 950 cGy in two divided doses. Chimeric mice reconstituted with bone marrow cells derived from either *miR-31*^fl/fl^ or cKO were subjected for EAE induction 8 weeks after the transplantation.

### Histology

Spinal cords from *miR-31*^*fl/fl*^ or cKO EAE mice were fixed in 4% paraformaldehyde and paraffin embedded. Paraffin-embedded 5-μm sections of spinal cord were stained with haematoxylin and eosin or Luxol fast blue and then examined by light microscopy (Axio scope A1, Zeiss).

### Luciferase reporter plasmid

The Gprc5a 3′-UTR was amplified using primers Gprc5a Forward, 5′- AATCTCGAGCTGTTGGGAAGAGTGGGAC -3′, Reverse, 5′- TCGGCGGCCGCAATAGTTGTGACCACATCTTTATTG -3′. The Gprc5a 3′-UTR genomic fragment was digested with XhoI-NotI and inserted into the corresponding sites of the psiCheck-2 Synthetic firefly luciferase reporter plasmid (Promega). This construct was also used to generate a miR-31 ‘seed' mutant plasmid. The mutagenic primers used for Gprc5a were Mutant Forward, 5′- AATCTCGAGCTGTTGGGAAGAGTGGGAC -3′, Mutant Reverse, 5′- CAGCCCCACCGTTCTCGGCGGTG -3′. The correctness of all the plasmids was confirmed by sequencing.

### Luciferase assays

All 3′ UTR reporter vectors were prepared by amplifying the 3′ UTRs of Gprc5a, followed by insertion into the psiCHECK-2 vector (Promega). Site-specific mutants were generated by PCR in the psiCHECK-2 vector (Promega). NIH3T3 cells were maintained in DMEM (HyClone) supplemented with 10% fetal bovine serum (HyClone), 2 mM glutamine, 100 IU ml^−1^ penicillin, 0.1 mg ml^−1^ streptomycin. NIH3T3 cells were seeded in the 24-well plates (1 × 10^5^ cells per well) one day before transfection and then each well was transfected with a mixture of 100 ng 3′-UTR luciferase reporter vector and 50 pmol miRNA mimics or controls. Twenty four hours post transfection, the cells were lysed. Then, luciferase activity was measured using the Dual-Luciferase Reporter Assay System (Promega), using a Lumat^3^ LB 9508 Single Tube Luminometer instrument (Berthold Technologies). Each experiment was performed in triplicate. The ratio of Renilla luciferase to Firefly luciferase was calculated for each well.

### Western blotting

Cultured T cells and the CNS of mice were lysed in radio immunoprecipitation assay buffer supplemented with protease and phosphatase inhibitor cocktail (Thermo Scientific). Mouse anti-actin Ab (1:3,000) (Cell Signaling Technology), rabbit anti-Gprc5a Ab (1:1,000) (Dr Jiong Deng provided) were used. The signal was detected with Pierce ECL Western Blotting Substrate (Thermo Scientific) and GE ImageQuant LAS 4000 (GE Healthcare). Images have been cropped for presentation. Full size images are presented in Supplementary Fig. 8.

### MicroRNA microarray analysis

Naive T cells from 3 *miR-31*^*fl/fl*^ mice and 3 *miR-31*^*fl/fl*^*CD4*^*Cre*^ mice were used to induce iT_reg_ *in vitro*. Total RNA was isolated using RNeasy MiniKit (Qiagen). Mouse genome-wide cDNA microarray analysis was performed by Shanghai Biotechnology (Shanghai).

### Statistical analysis

The data were analysed with GraphPad Prism 5 and were presented as the mean±s.e.m. Student's *t*-test was used when two conditions were compared, and analysis of variance with Bonferroni or Newman–Keuls correction was used for multiple comparisons. Probability values of <0.05 were considered significant; two-sided tests were performed.

## Additional information

**Accession codes**: Microarray data have been deposited in the GEO database under accession code GSE61938.

**How to cite this article:** Zhang, L. *et al*. MicroRNA-31 negatively regulates peripherally derived regulatory T-cell generation by repressing retinoic acid-inducible protein 3. *Nat. Commun.* 6:7639 doi: 10.1038/ncomms8639 (2015).

## Supplementary Material

Supplementary Information Supplementary Figures 1-8 and Supplementary Table 1

## References

[b1] Yamane H. & Paul W. E. Cytokines of the gamma(c) family control CD4+ T cell differentiation and function. Nat. Immunol. 13, 1037–1044 (2012). 23080204 10.1038/ni.2431 PMC4825860

[b2] Zhu J., Yamane H. & Paul W. E. Differentiation of effector CD4 T cell populations (*). Annu. Rev. Immunol. 28, 445–489 (2010). 20192806 10.1146/annurev-immunol-030409-101212 PMC3502616

[b3] Simmons S. B., Pierson E. R., Lee S. Y. & Goverman J. M. Modeling the heterogeneity of multiple sclerosis in animals. Trends Immunol. 34, 410–422 (2013). 23707039 10.1016/j.it.2013.04.006 PMC3752929

[b4] Fontenot J. D., Gavin M. A. & Rudensky A. Y. Foxp3 programs the development and function of CD4+CD25+ regulatory T cells. Nat. Immunol. 4, 330–336 (2003). 12612578 10.1038/ni904

[b5] Sakaguchi S., Miyara M., Costantino C. M. & Hafler D. A. FOXP3+ regulatory T cells in the human immune system. Nat. Rev. Immunol. 10, 490–500 (2010). 20559327 10.1038/nri2785

[b6] Abbas A. K. . Regulatory T cells: recommendations to simplify the nomenclature. Nat. Immunol. 14, 307–308 (2013). 23507634 10.1038/ni.2554

[b7] Curotto de Lafaille M. A. & Lafaille J. J. Natural and adaptive foxp3+ regulatory T cells: more of the same or a division of labor? Immunity 30, 626–635 (2009). 19464985 10.1016/j.immuni.2009.05.002

[b8] Chen W. . Conversion of peripheral CD4+CD25- naive T cells to CD4+CD25+ regulatory T cells by TGF-beta induction of transcription factor Foxp3. J. Exp. Med. 198, 1875–1886 (2003). 14676299 10.1084/jem.20030152 PMC2194145

[b9] Gottschalk R. A., Corse E. & Allison J. P. TCR ligand density and affinity determine peripheral induction of Foxp3 in vivo. J. Exp. Med. 207, 1701–1711 (2010). 20660617 10.1084/jem.20091999 PMC2916126

[b10] Venken K. . Compromised CD4+ CD25(high) regulatory T-cell function in patients with relapsing-remitting multiple sclerosis is correlated with a reduced frequency of FOXP3-positive cells and reduced FOXP3 expression at the single-cell level. Immunology 123, 79–89 (2008). 17897326 10.1111/j.1365-2567.2007.02690.x PMC2433271

[b11] Huan J. . Decreased FOXP3 levels in multiple sclerosis patients. J. Neurosci. Res. 81, 45–52 (2005). 15952173 10.1002/jnr.20522

[b12] Carbone F. . Regulatory T cell proliferative potential is impaired in human autoimmune disease. Nat. Med. 20, 69–74 (2014). 24317118 10.1038/nm.3411

[b13] Buckner J. H. Mechanisms of impaired regulation by CD4(+)CD25(+)FOXP3(+) regulatory T cells in human autoimmune diseases. Nat. Rev. Immunol. 10, 849–859 (2010). 21107346 10.1038/nri2889 PMC3046807

[b14] Kim V. N. & Nam J. W. Genomics of microRNA. Trends Genet. 22, 165–173 (2006). 16446010 10.1016/j.tig.2006.01.003

[b15] Ceribelli A., Satoh M. & Chan E. K. MicroRNAs and autoimmunity. Curr. Opin. Immunol. 24, 686–691 (2012). 22902047 10.1016/j.coi.2012.07.011 PMC3508200

[b16] Rossi R. L. . Distinct microRNA signatures in human lymphocyte subsets and enforcement of the naive state in CD4+ T cells by the microRNA miR-125b. Nat. Immunol. 12, 796–803 (2011). 21706005 10.1038/ni.2057

[b17] Cobb B. S. . A role for Dicer in immune regulation. J. Exp. Med. 203, 2519–2527 (2006). 17060477 10.1084/jem.20061692 PMC2118134

[b18] Liston A., Lu L. F., O'Carroll D., Tarakhovsky A. & Rudensky A. Y. Dicer-dependent microRNA pathway safeguards regulatory T cell function. J. Exp. Med. 205, 1993–2004 (2008). 18725526 10.1084/jem.20081062 PMC2526195

[b19] Chong M. M., Rasmussen J. P., Rudensky A. Y. & Littman D. R. The RNAseIII enzyme Drosha is critical in T cells for preventing lethal inflammatory disease. J. Exp. Med. 205, 2005–2017 (2008). 18725527 10.1084/jem.20081219 PMC2526196

[b20] Zhou X. . Selective miRNA disruption in T reg cells leads to uncontrolled autoimmunity. J. Exp. Med. 205, 1983–1991 (2008). 18725525 10.1084/jem.20080707 PMC2526194

[b21] Rouas R. . Human natural Treg microRNA signature: role of microRNA-31 and microRNA-21 in FOXP3 expression. Eur. J. Immunol. 39, 1608–1618 (2009). 19408243 10.1002/eji.200838509

[b22] Bastien J. & Rochette-Egly C. Nuclear retinoid receptors and the transcription of retinoid-target genes. Gene 328, 1–16 (2004). 15019979 10.1016/j.gene.2003.12.005

[b23] Hill J. A. . Retinoic acid enhances Foxp3 induction indirectly by relieving inhibition from CD4+CD44hi Cells. Immunity 29, 758–770 (2008). 19006694 10.1016/j.immuni.2008.09.018 PMC3140207

[b24] Ye X., Tao Q., Wang Y., Cheng Y. & Lotan R. Mechanisms underlying the induction of the putative human tumor suppressor GPRC5A by retinoic acid. Cancer Biol. Ther. 8, 951–962 (2009). 19279407 10.4161/cbt.8.10.8244

[b25] Park H. . A distinct lineage of CD4 T cells regulates tissue inflammation by producing interleukin 17. Nat. Immunol. 6, 1133–1141 (2005). 16200068 10.1038/ni1261 PMC1618871

[b26] Bilate A. M. & Lafaille J. J. Induced CD4+Foxp3+ regulatory T cells in immune tolerance. Annu. Rev. Immunol. 30, 733–758 (2012). 22224762 10.1146/annurev-immunol-020711-075043

[b27] Jager A., Dardalhon V., Sobel R. A., Bettelli E. & Kuchroo V. K. Th1, Th17, and Th9 effector cells induce experimental autoimmune encephalomyelitis with different pathological phenotypes. J. Immunol. 183, 7169–7177 (2009). 19890056 10.4049/jimmunol.0901906 PMC2921715

[b28] Thornton A. M. . Expression of Helios, an Ikaros transcription factor family member, differentiates thymic-derived from peripherally induced Foxp3+ T regulatory cells. J. Immunol. 184, 3433–3441 (2010). 20181882 10.4049/jimmunol.0904028 PMC3725574

[b29] Strainic M. G., Shevach E. M., An F., Lin F. & Medof M. E. Absence of signaling into CD4(+) cells via C3aR and C5aR enables autoinductive TGF-beta1 signaling and induction of Foxp3(+) regulatory T cells. Nat. Immunol. 14, 162–171 (2013). 23263555 10.1038/ni.2499 PMC4144047

[b30] Korn T. . IL-6 controls Th17 immunity *in vivo* by inhibiting the conversion of conventional T cells into Foxp3+ regulatory T cells. Proc. Natl Acad. Sci. USA 105, 18460–18465 (2008). 19015529 10.1073/pnas.0809850105 PMC2587589

[b31] Haribhai D. . A central role for induced regulatory T cells in tolerance induction in experimental colitis. J. Immunol. 182, 3461–3468 (2009). 19265124 10.4049/jimmunol.0802535 PMC2763205

[b32] Curotto de Lafaille M. A. . Adaptive Foxp3+ regulatory T cell-dependent and -independent control of allergic inflammation. Immunity 29, 114–126 (2008). 18617425 10.1016/j.immuni.2008.05.010

[b33] Tao Q., Cheng Y., Clifford J. & Lotan R. Characterization of the murine orphan G-protein-coupled receptor gene Rai3 and its regulation by retinoic acid. Genomics 83, 270–280 (2004). 14706456 10.1016/s0888-7543(03)00237-4

[b34] Robbins M. J. . Molecular cloning and characterization of two novel retinoic acid-inducible orphan G-protein-coupled receptors (GPRC5B and GPRC5C). Genomics 67, 8–18 (2000). 10945465 10.1006/geno.2000.6226

[b35] Tao Q. . Identification of the retinoic acid-inducible Gprc5a as a new lung tumor suppressor gene. J. Natl Cancer Inst. 99, 1668–1682 (2007). 18000218 10.1093/jnci/djm208

[b36] McGeachy M. J., Stephens L. A. & Anderton S. M. Natural recovery and protection from autoimmune encephalomyelitis: contribution of CD4+CD25+ regulatory cells within the central nervous system. J. Immunol. 175, 3025–3032 (2005). 16116190 10.4049/jimmunol.175.5.3025

[b37] O'Connor R. A., Malpass K. H. & Anderton S. M. The inflamed central nervous system drives the activation and rapid proliferation of Foxp3+ regulatory T cells. J. Immunol. 179, 958–966 (2007). 17617587 10.4049/jimmunol.179.2.958

[b38] Mekala D. J., Alli R. S. & Geiger T. L. IL-10-dependent infectious tolerance after the treatment of experimental allergic encephalomyelitis with redirected CD4+CD25+ T lymphocytes. Proc. Natl Acad. Sci. USA 102, 11817–11822 (2005). 16087867 10.1073/pnas.0505445102 PMC1188008

[b39] Viglietta V., Baecher-Allan C., Weiner H. L. & Hafler D. A. Loss of functional suppression by CD4+CD25+ regulatory T cells in patients with multiple sclerosis. J. Exp. Med. 199, 971–979 (2004). 15067033 10.1084/jem.20031579 PMC2211881

[b40] Yamane H. & Paul W. E. Early signaling events that underlie fate decisions of naive CD4+ T cells toward distinct T-helper cell subsets. Immunol. Rev. 252, 12–23 (2013). 23405892 10.1111/imr.12032 PMC3578301

[b41] Kanno Y., Vahedi G., Hirahara K., Singleton K. & O'Shea J. J. Transcriptional and epigenetic control of T helper cell specification: molecular mechanisms underlying commitment and plasticity. Annu. Rev. Immunol. 30, 707–731 (2012). 22224760 10.1146/annurev-immunol-020711-075058 PMC3314163

[b42] Molinero L. L., Miller M. L., Evaristo C. & Alegre M.-L. High TCR stimuli prevent induced regulatory T cell differentiation in a NF-κB–dependent manner. J. Immunol. 186, 4609–4617 (2011). 21411734 10.4049/jimmunol.1002361 PMC3544303

[b43] Lu L.-F. . Foxp3-dependent microRNA155 confers competitive fitness to regulatory T cells by targeting SOCS1 protein. Immunity 30, 80–91 (2009). 19144316 10.1016/j.immuni.2008.11.010 PMC2654249

[b44] Lu L.-F. . Function of miR-146a in controlling treg cell-mediated regulation of Th1 responses. Cell 142, 914–929 (2010). 20850013 10.1016/j.cell.2010.08.012 PMC3049116

[b45] de Kouchkovsky D. . microRNA-17–92 regulates IL-10 production by regulatory T cells and control of experimental autoimmune encephalomyelitis. J. Immunol. 191, 1594–1605 (2013). 23858035 10.4049/jimmunol.1203567 PMC4160833

[b46] Takahashi H. . TGF-[beta] and retinoic acid induce the microRNA miR-10a, which targets Bcl-6 and constrains the plasticity of helper T cells. Nat. Immunol. 13, 587–595 (2012). 22544395 10.1038/ni.2286 PMC3499969

[b47] Zhu J. . Down-regulation of Gfi-1 expression by TGF-beta is important for differentiation of Th17 and CD103+ inducible regulatory T cells. J. Exp. Med. 206, 329–341 (2009). 19188499 10.1084/jem.20081666 PMC2646571

[b48] Valastyan S. & Weinberg R. A. miR-31: a crucial overseer of tumor metastasis and other emerging roles. Cell Cycle 9, 2124–2129 (2010). 20505365 10.4161/cc.9.11.11843

[b49] Peng H. . microRNA-31/factor-inhibiting hypoxia-inducible factor 1 nexus regulates keratinocyte differentiation. Proc. Natl Acad. Sci. USA 109, 14030–14034 (2012). 22891326 10.1073/pnas.1111292109 PMC3435188

