# Supplementary material for: MicroRNA-31 negatively regulates peripherally derived regulatory T-cell generation by repressing retinoic acid-inducible protein 3
Source: Nat Commun. 2015 Jul 13;6:7639. doi: 10.1038/ncomms8639 (PMC4510656; doi:10.1038/ncomms8639)
Supplement: Supplementary Information — Supplementary Figures 1-8 and Supplementary Table 1 [file ncomms8639-s1.pdf]

Supplementary Information

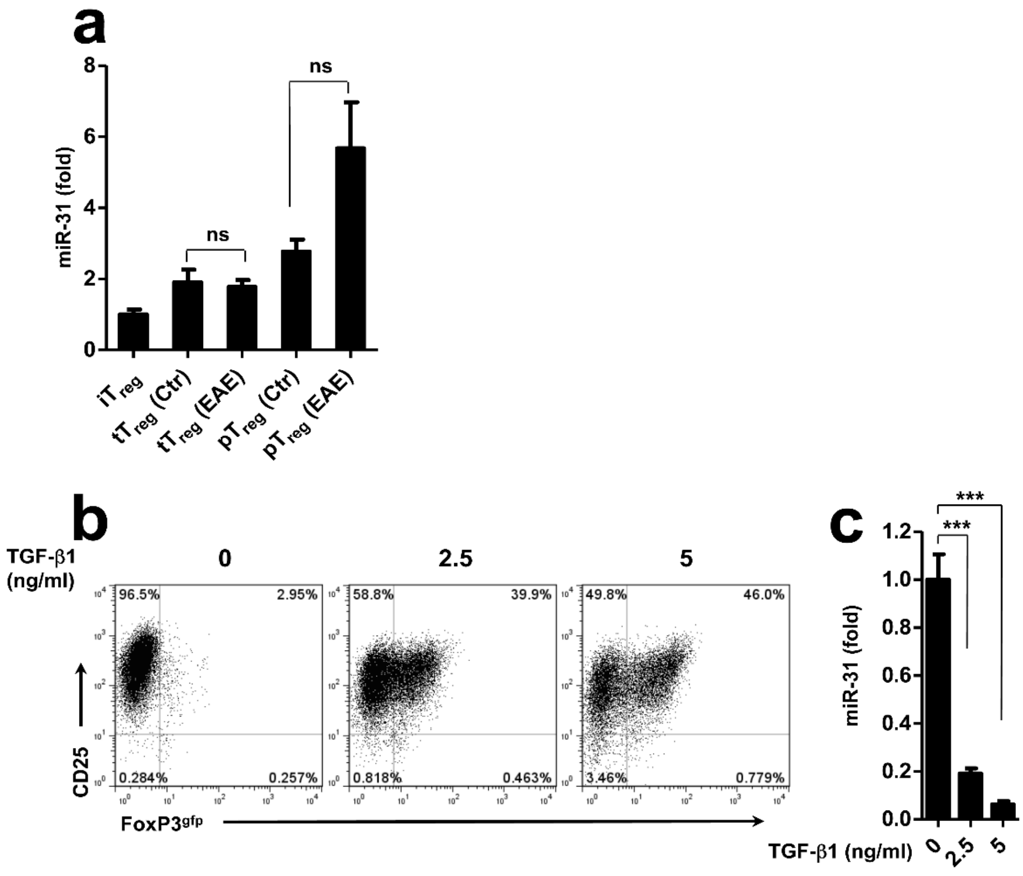

Supplementary Figure 1 miR-31 expression in T_{reg} subsets and its levels are reversely correlated with numbers of iT_{reg} cells. (**a**) miR-31 expression in iT_{reg}, tT_{reg}, EAE tT_{reg}, pT_{reg} and EAE pT_{reg} cells; results are presented relative to miR-31 expression in iT_{reg} cells. (**b**) Representative flow cytometry of $FoxP3^{gfp}$ expression in iT_{reg} cells polarized with different concentrations of TGF-β1. Numbers in quadrants indicate percent cells in each. (**c**) qPCR analysis of miR-31 expression in iT_{reg} cells polarized with different concentrations of TGF-β1; results are presented relative to miR-31 expression in naïve T cells cultured without TGF-β1. ns, not significant, ***$p<0.001$, two-tailed Student's *t*-test. Data are from one experiment representative of two independent experiments (mean ± sem).

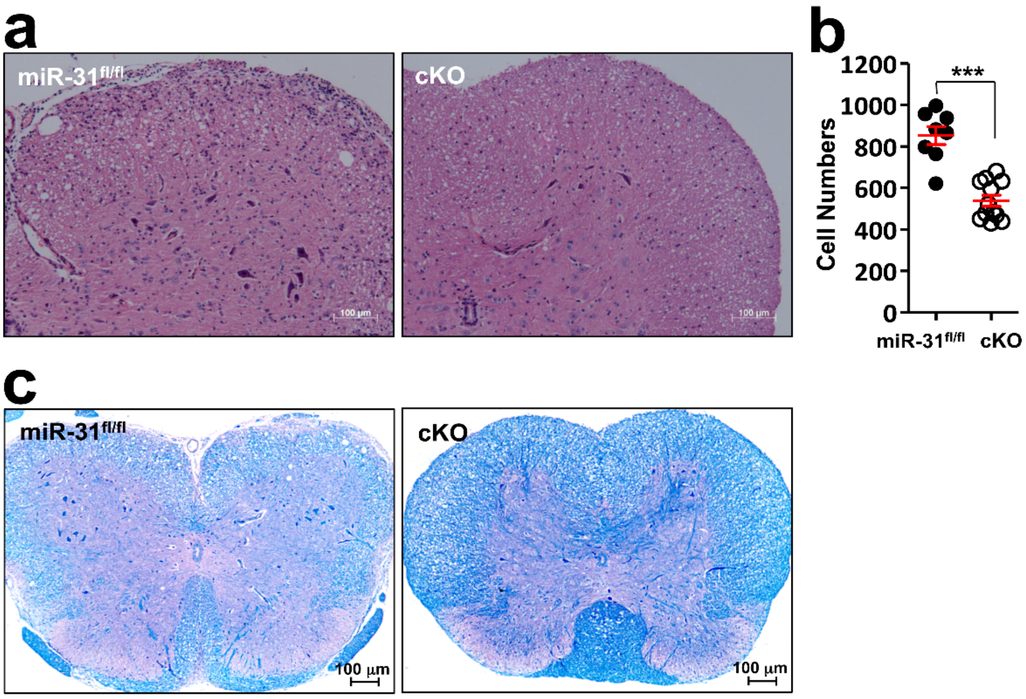

Supplementary Figure 2 Decreased infiltrating lymphocytes and demyelination of spinal cord in cKO mice with EAE. (**a, b**) Infiltrating lymphocytes in the spinal cord of *miR-31*$^{fl/fl}$ and cKO mice with EAE were counted. (**c**) The degree of demyelination of spinal cords from *miR-31*$^{fl/fl}$ and cKO mice with EAE was analyzed by Luxol fast blue staining. ****p*<0.001, two-tailed Student's *t*-test. Representative data are shown, which had been reproduced in two independent experiments.

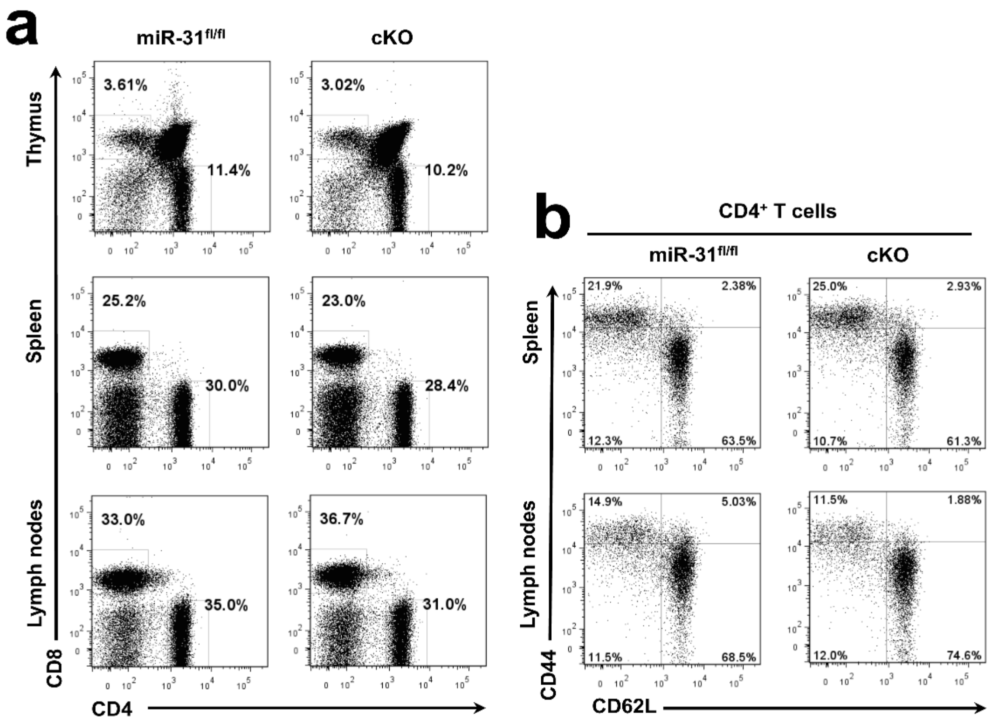

Supplementary Figure 3 miR-31 deficiency has no apparent effect on the development of T cells. (**a**) Representative flow cytometry of CD4$^+$ and CD8$^+$ T cells in thymus, spleen and lymph nodes from 6-week old *miR-31*$^{fl/fl}$ and cKO mice. (**b**) Representative flow cytometry for surface expression of CD44 and CD62L in CD4$^+$ cells in spleen and lymph nodes from 6-week old *miR-31*$^{fl/fl}$ and cKO mice. Numbers adjacent to outlined areas or in quadrants indicate percent cells in each. Data are from one experiment representative of two independent experiments.

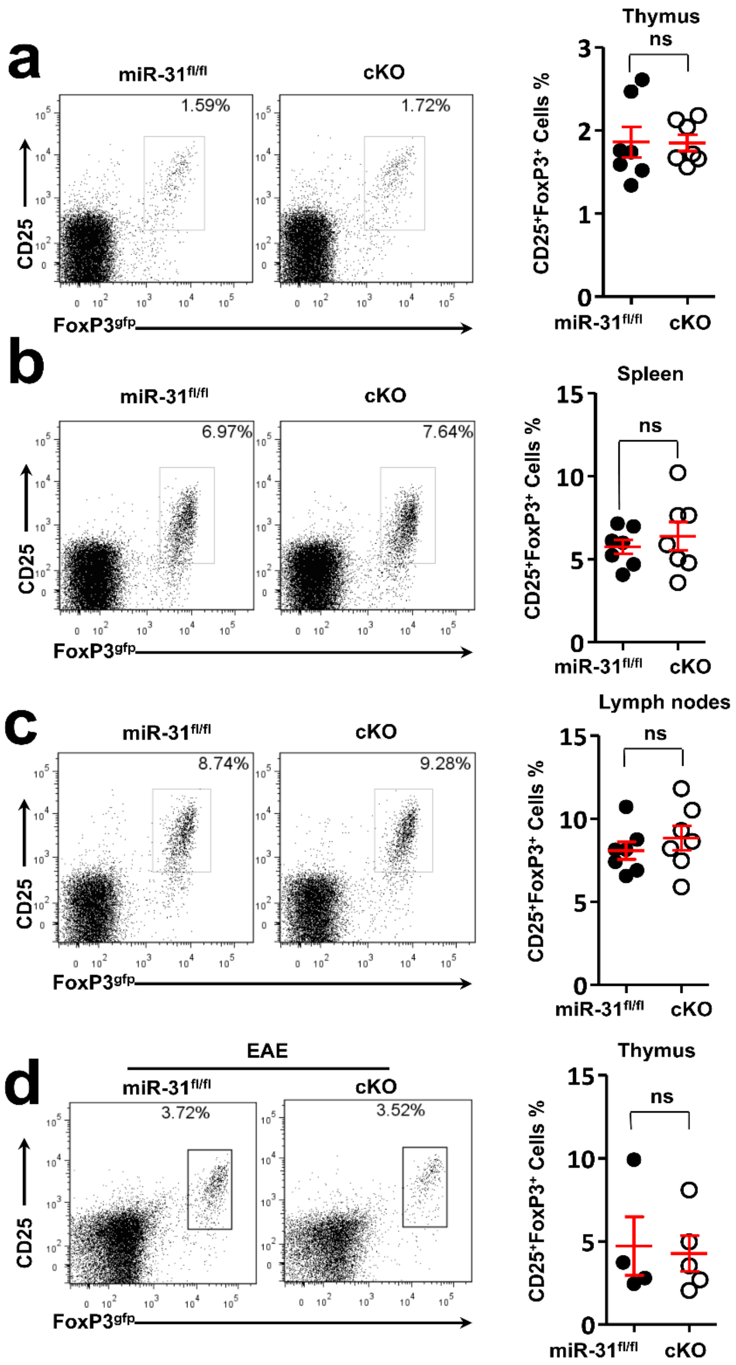

Supplementary Figure 4 miR-31 deficiency has no impact on tT$_{reg}$ cell development. *FoxP3^{gfp}* reporter mice were crossed with either *miR-31$^{fl/fl}$* or cKO mice. (**a-c**) Flow cytometric analysis of T$_{reg}$ cells in thymus, spleen and lymph nodes from 6-week old *miR-31$^{fl/fl}$* and cKO mice. (**d**) Flow cytometric analysis of tT$_{reg}$ cells in thymus from 6-week old *miR-31$^{fl/fl}$* and cKO mice with EAE. Numbers adjacent to outlined areas indicate percent cells in each. ns, not significant, two-tailed Student's *t*-test. Data are representative of three independent experiments (mean ± sem).

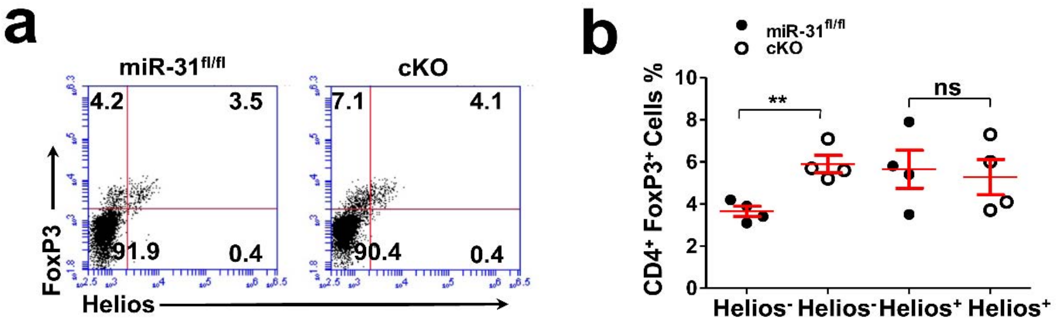

Supplementary Figure 5 Deletion of miR-31 in CD4$^+$ T cells promotes pT$_{reg}$ cell Induction *in vivo*. Bone marrow cells were prepared from either *miR-31*$^{fl/fl}$ or cKO mice. The bone marrow cells (5×10^6) were injected intravenously into lethally irradiated C57BL/6J recipient mice to generate bone marrow chimeric mice. Eight weeks after bone marrow transplantation, EAE was induced in all chimeric mice. (**a, b**) Splencytes prepared from *miR-31*$^{fl/fl}$ or cKO chimeric mice 10 days post immunization were subjected for analysis of Helios$^-$FoxP3$^+$ pT$_{reg}$ cells and Helios$^+$FoxP3$^+$ nT$_{reg}$ cells by Flow cytometry (cells were gated in CD4$^+$ T cell population). Data are representative of three independent experiments. **$p<0.01$, two-tailed *Student's test*.

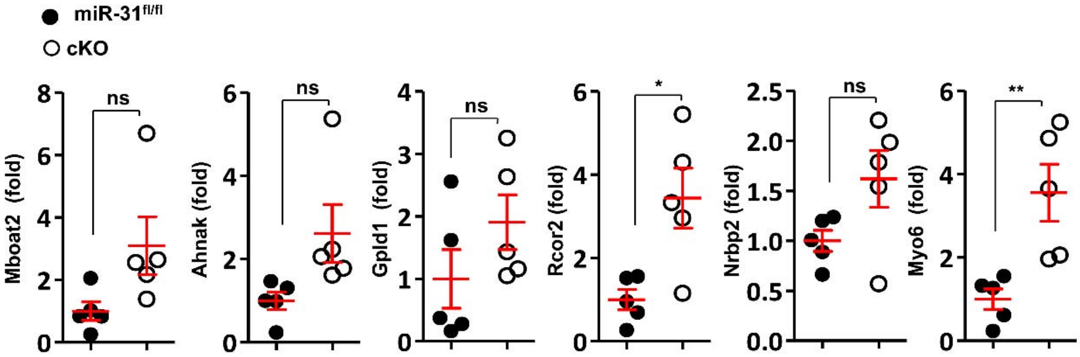

Supplementary Figure 6 mRNA levels of candidate genes targeted by miR-31 in iT_{reg} cells. mRNA levels of 6 candidate genes were measured by qPCR. Results are presented as the ratio of mRNA to β-actin, relative to that in *miR-31*$^{fl/fl}$ controls. *$p<0.05$, **$p<0.01$, ns, not significant, two-tailed Student's *t*-test. Data are representative of two independent experiments (mean ± sem).

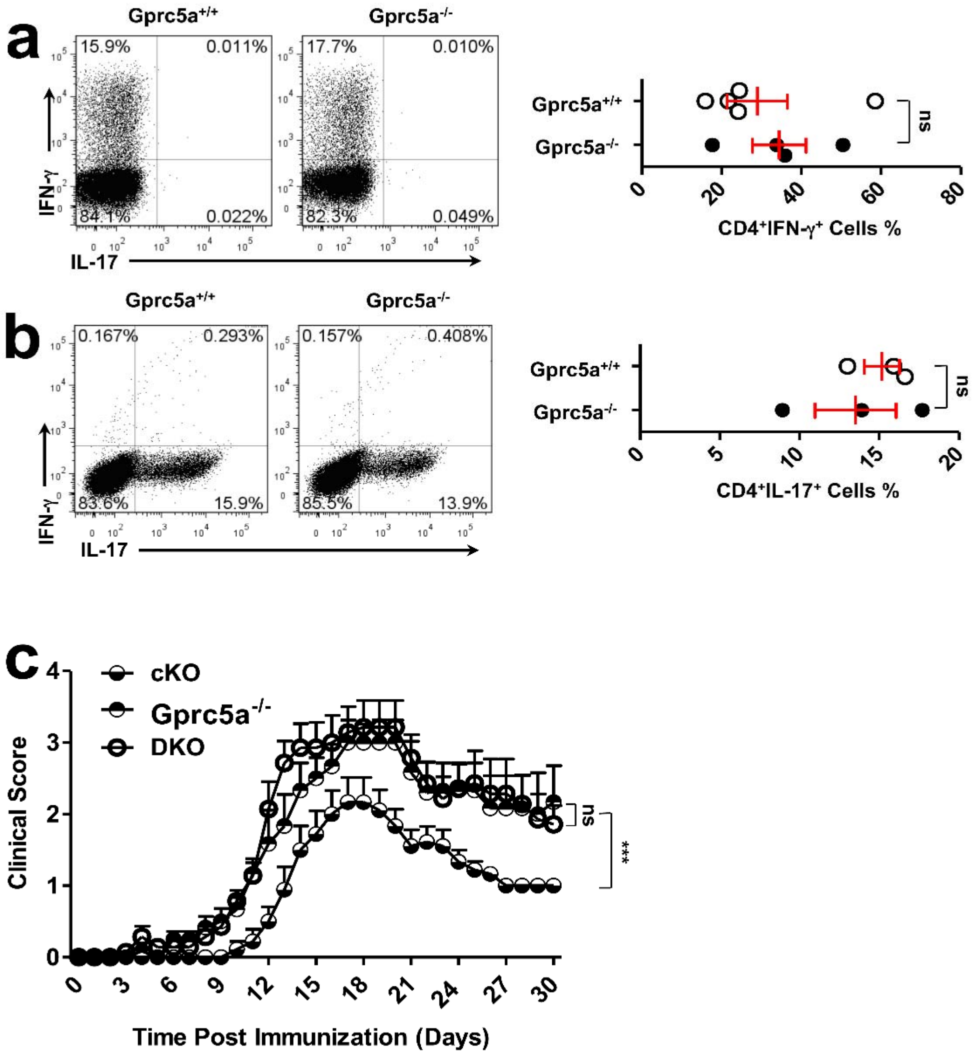

Supplementary Figure 7 Gprc5a deficiency does not affect T_H1 and T_H17 cell differentiation *in vitro*, but restores the disease phenotype in cKO mice. (**a, b**) Flow cytometric analysis of T_H1 and T_H17 cells polarized from naïve T cells of either *Gprc5a*$^{+/+}$ or *Gprc5a*$^{-/-}$ mice (n=3-4). Numbers in quadrants indicate percent cells in each. (**c**) miR-31 mice were crossed with *Gprc5a*$^{-/-}$ mice to generate DKO mice. Clinical scores (mean ± sem) of *cKO, Gprc5a*$^{-/-}$ *and DKO* mice after the induction of EAE were assessed every day (n=6-8). ***p<0.001, ns, not significant, two-tailed Student's *t*-test for (**a, b**), one-way ANOVA for (**c**). Data are from one representative of two independent experiments for (**a, b**) (mean ± sem).

Fig 5.e

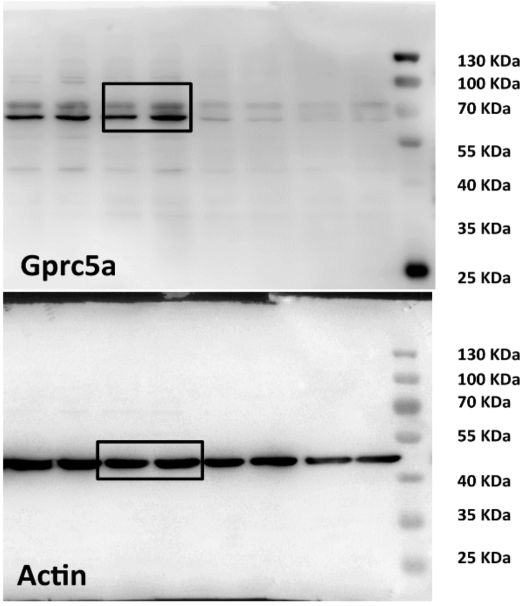

Fig 6.c

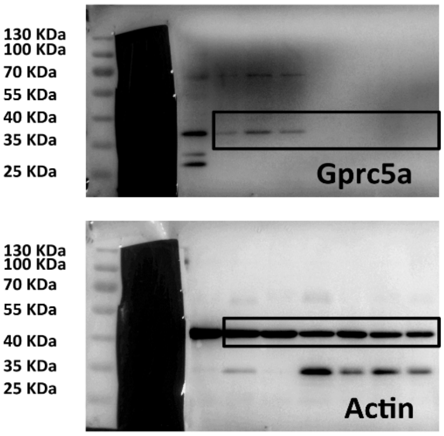

Fig 6.d

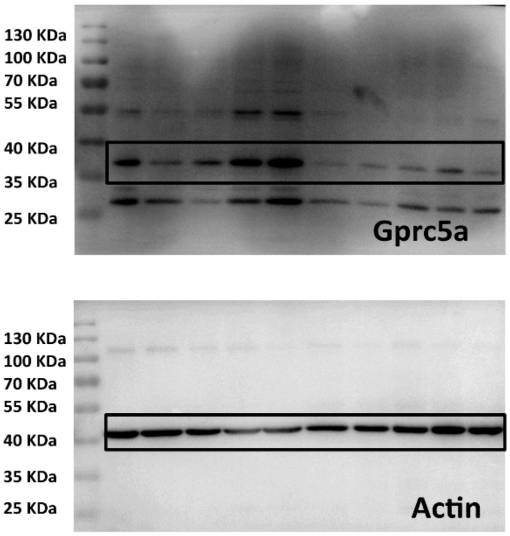

Supplementary Figure 8 List of original pictures of western blots. Black boxes highlight the indicated lanes in figures.

Supplementary Table 1

Primers used in this study

Molecule	Primer	Sequence
**FoxP3**	Upstream	CCCAGGAAAGACAGCAACCTT
	Downstream	TTCTCACAACCAGGCCACTTG
**b-actin**	Upstream	TGGAATCCTGTGGCATCCATGAAAC
	Downstream	TAAAACGCAGCTCAGTAACAGTCCG
**Mboat2**	Upstream	TCAGACACGTAGTTGCTACCC
	Downstream	TGCAGTAGGAAATCCCACTTTG
**Ahnak**	Upstream	CAGCGCATCTACACCACGAA
	Downstream	CACTTCATGCCTTGGTATCTTGA
**Gpld1**	Upstream	TATTCGAGAGAACTACCCTCTGC
	Downstream	AGGAACCCTTGTTCAATACCCA
**Rcor2**	Upstream	ATCCGAGTTGGAACCAATTACC
	Downstream	AGTGCCTGCTCAATGTTATAGC
**Gprc5a**	Upstream	ACCACAGACTTTGTGACCTGG
	Downstream	CGAGTGCAAACATGCAAGCC
**Myo6**	Upstream	CCACAATGTCAAAGTTCGGTACA
	Downstream	GGCATCGTCCCAAGAGATTTTC
**Nrbp2**	Upstream	AACGGGATCTATCCACTGATGA
	Downstream	GGTCTCCGAGTCAAAGGGTTC
**miR-31 -344~458**	Upstream	5'-TGCACGGGAGCATTCATACA
	Downstream	5'-GGATCCAATGGCGTTCAAGC
**miR-31 -531~795**	Upstream	TCTCCTTTCCTCACCCCACT
	Downstream	GACATGCGCTTTCCCAATCC
**miR-31-1175~1274**	Upstream	GTGACATGTTTGACTGCCGA
	Downstream	TGGCTCTGACTCATGAACTCC
**miR-31-1800~1936**	Upstream	TGTTCCGTTCACAAGCCCAT
	Downstream	AGGCTTTGATCCAGGCAGAC
